# Participants' outcomes gone missing within a network of interventions: Bayesian modeling strategies

**DOI:** 10.1002/sim.8207

**Published:** 2019-05-27

**Authors:** Loukia M. Spineli, Chrysostomos Kalyvas, Konstantinos Pateras

**Affiliations:** ^1^ Midwifery Research and Education Unit Hannover Medical School Hannover Germany; ^2^ Department of Biostatistics and Research Decision Sciences, MSD Europe Inc Brussels Belgium; ^3^ Department of Biostatistics and Research Support, Julius Center for Health Sciences and Primary Care University Medical Center Utrecht Utrecht The Netherlands

**Keywords:** Bayesian methods, missing outcome data, network meta‐analysis, pattern‐mixture model, simulation study

## Abstract

**Objectives:** To investigate the implications of addressing informative missing binary outcome data (MOD) on network meta‐analysis (NMA) estimates while applying the missing at random (MAR) assumption under different prior structures of the missingness parameter.

**Methods:** In three motivating examples, we compared six different prior structures of the informative missingness odds ratio (IMOR) parameter in logarithmic scale under pattern‐mixture and selection models. Then, we simulated 1000 triangle networks of two‐arm trials assuming informative MOD related to interventions. We extended the Bayesian random‐effects NMA model for binary outcomes and node‐splitting approach to incorporate these 12 models in total. With interval plots, we illustrated the posterior distribution of log OR, common between‐trial variance (*τ*^2^), inconsistency factor and probability of being best per intervention under each model.

**Results:** All models gave similar point estimates for all NMA estimates regardless of simulation scenario. For moderate and large MOD, intervention‐specific prior structure of log IMOR led to larger posterior standard deviation of log ORs compared to trial‐specific and common‐within‐network prior structures. Hierarchical prior structure led to slightly more precise *τ*^2^ compared to identical prior structure, particularly for moderate inconsistency and large MOD. Pattern‐mixture and selection models agreed for all NMA estimates.

**Conclusions:** Analyzing informative MOD assuming MAR with different prior structures of log IMOR affected mainly the precision of NMA estimates. Reviewers should decide in advance on the prior structure of log IMOR that best aligns with the condition and interventions investigated.

## 1 INTRODUCTION

Plenty of empirical studies on reporting quality of systematic reviews with conventional meta‐analyses have revealed several shortcomings in the reporting and administration of missing binary outcome data (MOD).^1^, ^2^, ^3^, ^4^ Recommendations aiming to improve reporting of systematic reviews with regards to MOD already exist and are built upon this comprehensive empirical evidence. Contrariwise, proposed guidelines for the administration of MOD in systematic reviews have evolved in the absence of simulation studies using only intuitive argumentations^5^, ^6^; for example, in the Cochrane Handbook, it is stated that “[imputing the missing data with replacement values] fails to acknowledge uncertainty in the imputed values and results, typically, in confidence intervals that are too narrow” (see chapter 16.1.2 in the work of Higgins and Green^6^). Current directions to deal with MOD in systematic reviews include (i) analysis of observed outcomes as a primary analysis, (ii) imputation of MOD under plausible scenarios as a sensitivity analysis, and (iii) statistical modeling of missingness mechanisms (ie, reasons that triggered MOD).^6^ The first two options are the most commonly adopted in systematic reviews.^1^, ^2^, ^3^ Nonetheless, they have been criticized for being employed inefficiently through data elimination or augmentation before analysis, respectively, and hence for ignoring the uncertainty induced by the scenarios considered.^6^, ^7^, ^8^ In turn, these options may compromise the conclusions of the systematic review.^9^

Statistical modeling of MOD has received little attention in systematic reviews with two (for example, the works of Ejere et al,^10^ Mayo‐Wilson et al,^11^ and Virgili et al^12^) or more interventions (for example, the works of Watt et al^13^ and Veroniki et al^14^). As opposed to imputation or exclusion, modeling MOD comprises an elegant framework that adjusts for bias due to MOD and fully acknowledges the uncertainty about the scenarios considered for the missingness mechanism. This is achieved by modeling the joint distribution of the outcomes (observed and missing) and missingness indicator.^15^ This joint distribution is further factorized in two ways: a distribution of the outcome, given the missingness indicator, and a distribution of that indicator (pattern‐mixture model)^16^ or a distribution of the missingness indicator, given the underlying outcome, and a distribution of the underlying outcome (selection model).^17^ Selection model is more prevalent in the literature for clinical trials,^18^ while pattern‐mixture model has been most frequently described in the analysis of series of trials.^19^ Modeling MOD using either pattern‐mixture or selection models offers a thorough investigation of the underlying missingness mechanisms across different trials and interventions.^8^, ^20^, ^21^ These mechanisms can be naturally explored using Bayesian approaches, where the reviewer assigns an informative prior distribution on the missingness parameter (ie, an absolute or relative measure of the relationship between outcome and missingness indicator) to indicate a specific scenario alongside the uncertainty for that scenario.^8^

The existing directions on reporting and handling MOD in conventional systematic reviews are of great relevance and importance also for systematic reviews with network meta‐analysis (NMA). NMA offers an in‐depth exploration of the missingness mechanisms in the network as interventions may carry a different degree of and reasons for MOD in different comparisons and this information cannot be located in isolated conventional meta‐analyses. Moreover, due to the addition of interventions, assumptions, and model parameters that structure this framework, addressing MOD in NMA can reveal their implications on model parameters beyond the standard meta‐analytic ones. Since the statistical methodology of NMA has been refined and implemented mainly within the Bayesian framework,^22^, ^23^, ^24^ we view statistical modeling with the assignment of carefully selected prior distribution on the missingness parameter as a natural way to handle MOD in a network of interventions.

To our knowledge, there is currently no published empirical or simulation study on the comparative performance of models for MOD using Bayesian approaches in terms of meta‐analysis or NMA estimates. Consequently, the analyst misses the knowledge of the overall performance of models for aggregated MOD to critically decide on the proper models to apply. To shed light on this knowledge gap, we set up a comprehensive simulation study using empirical evidence from published NMAs in a wide range of health‐related fields to inform the simulation setting for a triangle network of two‐arm trials. This simulation study aims to designate the factors that may affect the performance of modeling informative MOD (ie, the missingness mechanism depends on the unobserved outcomes^25^) on the basis of core NMA estimates while assuming missing at random (MAR) for analysis as a starting point.^7^, ^20^, ^26^ Furthermore, the simulation results supplement the observations from a relevant empirical study^27^ in order to provide empirically‐based recommendations for a proper modeling of MOD in systematic reviews.

This article is organized as follows. In Section 2, we present the Bayesian random‐effects NMA model for binary outcomes in the absence of MOD (as described by Dias et al^28^), and then, we expand the model to incorporate MOD through pattern‐mixture and selection models.^8^, ^21^ Then, we present the prior structures for the missingness parameter that we considered in the simulation study. In Section 3, we illustrate these prior structures under pattern‐mixture and selection models in three published systematic reviews with NMA. In Section 4, we describe a novel simulation setup that combines already established data generation models for conventional meta‐analysis with specific algorithms to incorporate MOD in NMA, and we present the results in Section 5; in Section 6, we discuss the findings and limitations of the study and we provide recommendations, and we conclude in Section 7.

## 2 MISSING OUTCOME DATA IN NETWORK META‐ANALYSIS

### 2.1 Bayesian random‐effects NMA model

Consider a network of *N* trials that investigate different sets of *T* interventions for a specific condition. The outcome of interest is binary and the frequency of outcome in arm *k* = 1, 2, …, *a*_*i*_ of trial *i* = 1, 2, …, *N* is assumed to be a realization from the binomial distribution

$$r_{i,k}∼\text{Bin}\left(p_{i,k},,,n_{i,k}\right),$$

with *p*_*i*,*k*_ being the underlying risk of an event (the parameter of interest) and *n*_*i*,*k*_ the randomized sample in arm *k* of trial *i*. Then, using a logit function, as described by Dias et al,^28^ the log odds of event in arm *k* of trial *i* are defined as follows:

(1) $$\text{logit}\left(p_{i,k}\right)=u_{i}+\theta_{i,k1},$$

*u*_*i*_ = logit(*p*_*i*,1_) is the log odds of event in the baseline arm of trial *i* and *θ*_*i*,*k*1_ is the log odds ratio (OR) of event in arm *k* relative to the baseline arm that typically follows a normal distribution with mean $\mu_{t_{i,k}t_{i,1}}$ and variance *τ*^2^ commonly assumed to be constant across different comparisons. Index *t*_*i*,*k*_ indicates the intervention studied in arm *k* of trial *i*.

#### 2.1.1 Incorporating multi‐arm trials

In a trial *i* with *a*_*i*_ > 2 arms, log ORs are correlated since they share the same comparator, and therefore, the vector ***θ***_*i*_ of *a*_*i*_ − 1 log ORs follows a multivariate normal distribution^28^, ^29^

$$\theta_{i}=\left(\begin{array}{c} \theta_{i,21}\\ \vdots \\ \theta_{i,a_{i}1} \end{array}\right)∼{\text{MVN}}_{a_{i}-1}\left(\left(\begin{array}{c} \mu_{t_{i,2}t_{i,1}}\\ \vdots \\ \mu_{t_{i,a_{i}}t_{i,1}} \end{array}\right),\left(\begin{array}{ccc} \begin{array}{c} \tau^{2}\\ \tau^{2}/2 \end{array} & \begin{array}{c} \tau^{2}/2\\ \tau^{2} \end{array} & \begin{array}{c} \text{⋯}\\ \text{⋯} \end{array}\begin{array}{c} \tau^{2}/2\\ \tau^{2}/2 \end{array}\\ \vdots & \vdots & \begin{array}{cc} \;\ddots & \;\vdots \end{array}\\ \tau^{2}/2 & \tau^{2}/2 & \begin{array}{cc} \;\text{⋯} & \;\tau^{2} \end{array} \end{array}\right)\right),$$

which, under the consistency assumption, is equivalent to conditional univariate normal distributions as follows^28^:

$$\theta_{i,k1}|\left(\begin{array}{c} \theta_{i,21}\\ \vdots \\ \theta_{i,\left(a_{i}-1\right)1} \end{array}\right)∼N\left(\left(\mu_{t_{i,k}A}-\mu_{t_{i,1}A}\right)+\frac{1}{a_{i}}\sum_{j=2}^{a_{i}-1}\left(\theta_{i,j1}-\left(\mu_{t_{i,j}A}-\mu_{t_{i,1}A}\right)\right),\frac{a_{i}}{2\cdot \left(a_{i}-1\right)}\cdot \tau^{2}\right),$$

where *μ*_*tA*_ reflects the relative treatment effects of the comparisons with the reference intervention of the network (known as basic parameters^30^), *A*. Then, using the consistency equation, the relative treatments effects of all possible nonreference comparisons can be obtained as functions of the basic parameters

$$\mu_{\text{tl}}=\mu_{\text{tA}}-\mu_{\text{lA}},$$

with *t*, *l* = {*B*, *C*, …, *T*} ∌ *A* and *t* ≠ *l*.

In the Bayesian framework, all parameters of the model are random variables that need proper prior distributions. In the present study, we used noninformative normal prior distribution with mean 0 and variance 10 000 for the location parameters (ie, *u*_*i*_ and *μ*_*tA*_), whereas we considered *HN*(0, 1) (median: 0.98, interquartile range [IQR]: 0.51‐1.96) as a weakly informative prior distribution on *τ* due to trial sparsity in the investigated networks that may compromise a proper estimation of *τ*.

#### 2.1.2 Rank probabilities for each intervention

To facilitate decision‐making, we can estimate for each intervention the probability of being first, second, third, and so on for a specific outcome.^31^ These rank probabilities are estimated by ordering the basic parameters in each iteration of the Markov chain Monte Carlo (MCMC) simulation and then, for each intervention, calculating the frequency to achieve a specific rank out of the number of iterations.

#### 2.1.3 Node‐splitting approach to assessing local inconsistency

To assess possible inconsistency locally while using the whole network to obtain an indirect effect for a comparison of a closed loop, Dias et al^32^ proposed the node‐splitting approach within a Bayesian framework. Specifically, a comparison from a closed loop is isolated (split) and random‐effects meta‐analysis is applied, whereas the remaining network is used to estimate an indirect effect for the split comparison. Then, the difference between direct and indirect effect for that comparison yields a posterior distribution for the inconsistency between these two effects, known as inconsistency factor (IF). A large posterior probability of IF being different from zero (eg, above 95%) provides sufficient evidence that inconsistency may be present in a specific loop. To improve the estimation of *τ*^2^, a common *τ*^2^ is assumed for both meta‐analysis and NMA model after removing the trials of the split comparison.

### 2.2 Modeling missing outcome data

#### 2.2.1 Pattern‐mixture model

Suppose that *m*_*i*,*k*_ participants were missing (for reasons related or not to the design and conduct of the trial) in arm *k* of trial *i* with probability *q*_*i*,*k*_, whereas among those $n_{i,k}^{o}=n_{i,k}-m_{i,k}$ participants who were observed, only $r_{i,k}^{o}$ experienced the studied outcome with probability $p_{i,k}^{o}$. It follows that the number of MOD and the number of observed events in arm *k* of trial *i* are realizations from the respective binomial distributions

$$m_{i,k}∼\text{Bin}\left(q_{i,k},,,n_{i,k}\right)\;\text{and}\;r_{i,k}^{o}∼\text{Bin}\left(p_{i,k}^{o},,,n_{i,k}^{o}\right).$$

In the presence of MOD, a pattern‐mixture model can be considered, where *p*_*i*,*k*_ is modeled conditional on whether the underlying event is observed or missing

(2) $$p_{i,k}=p_{i,k}^{o}\cdot \left(1-q_{i,k}\right)+p_{i,k}^{m}\cdot q_{i,k},$$

where $p_{i,k}^{m}$ is the missingness parameter and indicates the probability of event conditional on MOD in arm *k* of trial *i*. The parameters $p_{i,k}^{o}$ and *q*_*i*,*k*_ can be estimated directly from the data, whereas we need a proper prior distribution on $p_{i,k}^{m}$ to describe a plausible missingness mechanism.

Following the work of Turner et al,^8^ after rearranging Equation (2) to link $p_{i,k}^{o}$ with the remaining parameters, we obtain the following equation:

$$p_{i,k}^{o}=\frac{p_{i,k}-p_{i,k}^{m}\cdot q_{i,k}}{1-q_{i,k}}.$$

Subsequently, we use Equation (1) with a random‐effects model for *θ*_*i*,*k*1_ to apply the NMA model.

#### 2.2.2 Selection model

Instead of applying separate binomial distributions, we can jointly model all observed data via the following multinomial distribution^20^, ^21^:

$$L_{i,k}∼M\left(p_{1,i,k},p_{2,i,k},p_{3,i,k},n_{i,k}\right),$$

where ***L***_***i*,*k***_ is a vector of all data observed in arm *k* of trial *i*, namely, ${\left(r_{i,k}^{o},,,n_{i,k}-r_{i,k}^{o}-m_{i,k},,,m_{i,k}\right)}^{T}$ and

$$\;\begin{array}{cc} p_{1,i,k} & =\left(1-c_{1,i,k}\right)\cdot p_{i,k}\\ p_{2,i,k} & =\left(1-c_{0,i,k}\right)\cdot \left(1-p_{i,k}\right) \end{array}$$

(3) $$q_{i,k}=p_{3,i,k}=c_{1,i,k}\cdot p_{i,k}+c_{0,i,k}\cdot \left(1-p_{i,k}\right),$$

where *p*_1,*i*,*k*_ reflects the marginal probability of observing the underlying event, *p*_2,*i*,*k*_ reflects the marginal probability of observing the underlying nonevent, and *q*_*i*,*k*_ is the probability of MOD out of the randomized sample in arm *k* of trial *i*, respectively. The latter equation actually describes the *selection model* that has already been proposed in a conventional meta‐analysis^20^ and extended to operate in NMA.^21^ Then, parameters *c*_1,*i*,*k*_ and *c*_0,*i*,*k*_ indicate the probability of MOD conditional on those participants with the underlying event and the probability of MOD conditional on those participants without the underlying event, respectively. Apart from *q*_*i*,*k*_, all other parameters are not estimable from the data, and hence, we need to assign proper prior distributions for precise inference to be possible.

#### 2.2.3 Informative missingness odds ratio as missingness parameter

In the present study, we focus on the informative missingness odds ratio (IMOR) parameter, which, under the pattern‐mixture model, is defined as follows^7^, ^8^, ^33^:

$$\delta_{i,k}^{\text{PM}}=\frac{p_{i,k}^{m}/\left(1-p_{i,k}^{m}\right)}{p_{i,k}^{ο}/\left(1-p_{i,k}^{o}\right)},$$

while under the selection model, it is defined as^20^, ^21^

$$\delta_{i,k}^{S}=\frac{c_{1,i,k}/\left(1-c_{1,i,k}\right)}{c_{0,i,k}/\left(1-c_{0,i,k}\right)}.$$

Similar to OR, IMOR takes nonnegative values; nevertheless, due to different factorizations of the same joint distribution of outcome and missingness indicator under pattern‐mixture (PM) and selection (S) models, IMOR has different interpretation with respect to these models:

- $\delta_{i,k}^{\text{PM}}>1$, the odds of underlying event among those participants being missing is more likely than the odds of underlying event among those participants being observed in arm *k* of trial *i*;
- $\delta_{i,k}^{S}>1$, the odds of MOD among participants with underlying event is more likely than the odds of MOD among participants without underlying event in arm *k* of trial *i*;
- $\delta_{i,k}^{\text{PM}}<1$, the odds of underlying event among those participants being observed is more likely than the odds of underlying event among those participants being missing in arm *k* of trial *i*;
- $\delta_{i,k}^{S}<1$, the odds of MOD among participants without underlying event is more likely than the odds of MOD among participants with underlying event in arm *k* of trial *i*;
- $\delta_{i,k}^{\text{PM}}=1$, the outcome is similarly distributed between those participants being missing and those being observed in arm *k* of trial *i* (ie, MAR assumption);
- $\delta_{i,k}^{S}=1$, MOD are equally likely to occur among participants with underlying event and those without underlying event in arm *k* of trial *i* (ie, MAR assumption).

Like OR, IMOR is applied in the logarithmic scale but it is back‐transformed to facilitate in the interpretation

$$\begin{array}{cc} \log\left(\delta_{i,k}^{\text{PM}}\right) & =\varphi_{i,k}^{\text{PM}}=\text{logit}\left(p_{i,k}^{m}\right)-\text{logit}\left(p_{i,k}^{o}\right)\\ \log\left(\delta_{i,k}^{S}\right) & =\varphi_{i,k}^{S}=\text{logit}\left(c_{1,i,k}\right)-\text{logit}\left(c_{0,i,k}\right) \end{array}$$

under pattern‐mixture model and selection model, respectively.

#### 2.2.4 Structural assumptions to model informative missingness odds ratio

To investigate the underlying missingness mechanisms while acknowledging the uncertainty regarding our prior belief, normal prior distributions are assigned on $\varphi_{i,k}^{l}$ with carefully selected values for the mean ( $\mu_{i,k}^{\varphi }$) and variance ( $\sigma_{i,k}^{2}$) that reflect a plausible belief about the missingness mechanism on average and make $\varphi_{i,k}^{l}$ identifiable, respectively,

$$\varphi_{i,k}^{l}∼N\left(\mu_{i,k}^{\varphi },,,\sigma_{i,k}^{2}\right)\;\text{for}\;l=\text{PM},S.$$

Following the work of White et al,^20^ we considered $\varphi_{i,k}^{l}$'s to be on average MAR (as recommended by relevant published literature to address MOD in the primary analysis^7^, ^20^, ^26^) and exchangeable across trials and interventions, that is, $\mu_{i,k}^{\varphi }=0$ and $\sigma_{i,k}^{2}=\sigma^{2}$. White et al^20^, ^33^ recommended choosing *σ*^2^ ∈ [0.25, 4], which covers a range of values for log IMOR reflecting liberal to conservative uncertainty about the missingness scenario considered. In the present study, we used *σ*^2^ = 1:

(4) $$\varphi_{i,k}^{l}∼N\left(0,1\right)\;\text{for}\;l=\text{PM},S.$$

The prior distribution (4) can be shaped further to accommodate our prior beliefs regarding how different $\varphi_{i,k}^{l}$'s can be related within the network.^8^, ^20^ Following our empirical study,^27^ we considered identical and hierarchical prior structure for $\varphi_{i,k}^{l}$. Under identical structure, $\varphi_{i,k}^{l}$ is assumed to be the same across trials that investigate the same interventions but different across interventions (intervention‐specific)

$$\varphi_{i,k}^{l}=\varphi_{t_{\text{ik}}}^{l},\varphi_{t_{\text{ik}}}^{l}∼N\left(0,1\right)$$

or the same across interventions compared in a trial but different across trials (trial‐specific)

$$\varphi_{i,k}^{l}=\varphi_{i}^{l},\varphi_{i}^{l}∼N\left(0,1\right)$$

or identical across all trials and interventions (common‐within‐network)

$$\varphi_{i,k}^{l}=\varphi^{l},\varphi^{l}∼N\left(0,1\right).$$

Hierarchical structure “relaxes” the identical structure by assuming $\varphi_{i,k}^{l}$'s to be different yet related to each other. Then, intervention‐specific $\varphi_{i,k}^{l}$ under on average MAR is defined as

$$\varphi_{i,k}^{l}∼N\left(\mu_{t_{\text{ik}}}^{\varphi },,,\sigma_{t_{\text{ik}}}^{2}\right)\;\text{with}\;\mu_{t_{\text{ik}}}^{\varphi }∼N\left(0,1\right),\sigma_{t_{\text{ik}}}∼U\left(0,1\right);$$

trial‐specific $\varphi_{i,k}^{l}$ on average MAR is defined as

$$\varphi_{i,k}^{l}∼N\left(\mu_{i}^{\varphi },,,\sigma_{i}^{2}\right)\;\text{with}\;\mu_{i}^{\varphi }∼N\left(0,1\right)\;\text{and}\;\sigma_{i}∼U\left(0,1\right);$$

and common‐within‐network $\varphi_{i,k}^{l}$ on average MAR is defined as

$$\varphi_{i,k}^{l}∼N\left(\mu^{\varphi },,,\sigma^{2}\right)\;\text{with}\;\mu^{\varphi }∼N\left(0,1\right),\sigma ∼U\left(0,1\right).$$

We assigned a uniform distribution on *σ*, *σ*_*i*_, and $\sigma_{t_{\text{ik}}}$; however, other appropriate prior distributions for variance components can be also considered.^34^, ^35^

## 3 ILLUSTRATIVE EXAMPLES

Among the NMAs we retrieved in our previous study,^36^ we considered three NMAs with at least one closed loop: one with low MOD in the included trials; one with moderate MOD that are balanced within the trials; and one with moderate MOD that are unbalanced within the trials. Only one NMA had large MOD in the included trials^37^; however, it was a star‐shaped network, and therefore, we did not consider it in the present study. This classification of networks according to the amount of MOD is based on a decision rule we developed.^27^ A brief description of this decision rule is available as in Supporting Information (S.1).

We analyzed all three networks under pattern‐mixture and selection models using the prior structures for log IMOR described in Section 2.2.4. These networks included essentially different interventions (placebo and active interventions of different composition), and hence, we expected log IMORs to differ across the interventions as well as to be different yet related across the corresponding trials. Therefore, we considered hierarchical, intervention‐specific prior for log IMORs as the most plausible modeling strategy for the motivating examples. We used interval plots to present the results on the posterior distribution of log ORs for the basic parameters, *τ*^2^, and IFs for all 16 models considered, whereas we used rankograms to illustrate the rank probabilities of all interventions on all possible ranks. For each model, we used *HN*(0, 1) on *τ*. Two parallel chains with different initial values were used for 100 000 updates and a burn‐in of 10 000 MCMC samples.^38^ Convergence assessment was based on the Gelman‐Rubin convergence diagnostic, $\hat{R}$,^39^ and inspection of trace and autocorrelation plots. Initially, we used the R package *gemtc* 40, 41 to identify the comparisons to split in each network, and then, we inserted these comparisons in the node‐splitting model developed by Dias et al,^32^ which we expanded further to incorporate the IMOR parameter. The network plots were created with the R package *pcnetmeta*,^42^ while the figures illustrating the results were created with the R package *ggplot2*.^43^ All analyses were performed in the statistical software R version 3.3.1 using JAGS via the R package *R2jags*.^44^, ^45^

### 3.1 Example 1: low missing outcome data

**Figure 1:**
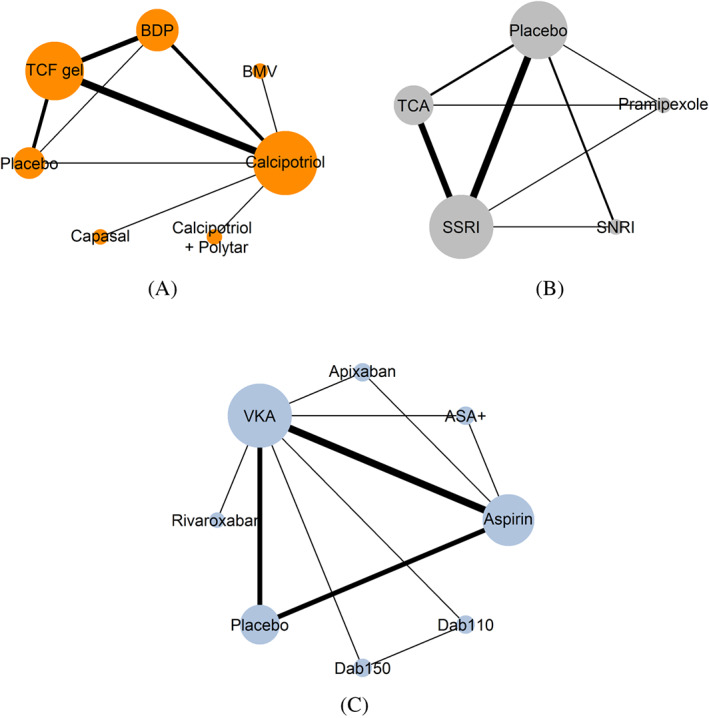
A series of network plots on (A) the effectiveness of topical therapies for moderately severe scalp psoriasis,^46^ (B) the efficacy of antidepressants in Parkinson's disease,^47^ and (C) the prevention of a stroke episode in patients with atrial fibrillation using oral antithrombotics.^48^ The thickness of the lines and the size of the nodes are proportional to the number of trials and the number of patients randomized in the respective treatments, respectively. ASA+, aspirin plus clopidogrel; Dab110, dabigatran 110 mg; Dab150, dabigatran 150 mg; BDP, betamethasone dipropionate; BMV, betamethasone valerate; SNRI, serotonin–norepinephrine reuptake inhibitor; SSRI, selective serotonin reuptake inhibitor; TCA, tricyclic antidepressant; TCF, two‐compound formulation; VKA, vitamin K antagonist [Colour figure can be viewed at wileyonlinelibrary.com]

Bottomley et al^46^ investigated the effectiveness of seven interventions measured as the investigator's global assessment response at 4 weeks in patients with moderately severe scalp psoriasis. A total of 9 trials (7 two‐arm, 1 three‐arm, and 1 four‐arm trials) with 5889 patients (median per trial: 237, IQR: 136‐419) formed the network (Figure 1A). For this outcome, MOD were low (median per trial: 3%, IQR: 1%‐6%) in the included trials. Positive log OR indicated a beneficial effect of the first intervention of the comparison.

Overall, results on log ORs were almost identical for all missingness models (pattern‐mixture or selection model) and prior structures of log IMOR (Supporting Information S.2; Figure S1). As a result, the ranking curves were indistinguishable for different prior structures of log IMOR in both missingness models (Supporting Information S.2; Figure S2). Results were also similar for *τ*^2^, although the 95% credible intervals (CrIs) were slightly narrower for hierarchical, trial‐specific prior structure of log IMORs in both missingness models (Supporting Information S.2; Figure S1). Results on node‐splitting were in line with those on basic parameters (Supporting Information S.2; Figure S3).

### 3.2 Example 2: moderate and balanced missing outcome data

Liu et al^47^ assessed the comparative effectiveness of four antidepressants and placebo in Parkinson's disease measured as the proportion of patients who had a reduction of at least 50% from the baseline score (Figure 1B). For this outcome, the authors included a total of 11 trials (8 two‐arm and 3 three‐arm trials) with 801 patients (median per trial: 19, IQR: 17‐33). MOD were moderate (median per trial: 16%, IQR: 12%‐24%) and balanced (median per trial: 4%, IQR: 2%‐11%) in the included trials. Positive log OR indicated beneficial effect of the first intervention of the comparison.

Results on log ORs were similar overall, albeit the 95% CrIs were slightly wider for (identical and hierarchical) intervention‐specific prior structure of log IMORs in both missingness models (Supporting Information S.3; Figure S4). Nevertheless, *τ*^2^ was slightly lower (and with slightly narrower 95% CrIs) for hierarchical as compared to identical prior structure of log IMOR regardless of further structural assumptions or missingness model. No profound differences were observed on rank probabilities (Supporting Information S.3; Figure S5) and the results from node‐splitting approach (Supporting Information S.3; Figure S6).

### 3.3 Example 3: moderate and unbalanced missing outcome data

Dogliotti et al^48^ assessed the comparative effectiveness of seven antithrombotic therapies and placebo in terms of preventing a stroke episode in patients with atrial fibrillation (Figure 1C). The authors included 16 trials (12 two‐arm and 4 three‐arm trials) with 79 808 patients (median per trial: 391, IQR: 211‐2940). MOD were moderate (median per trial: 19%, IQR: 13%‐23%) and slightly unbalanced (median per trial: 7%, IQR: 3%‐10%). Negative log OR indicated a beneficial effect of the first intervention in the comparison.

**Figure 2:**
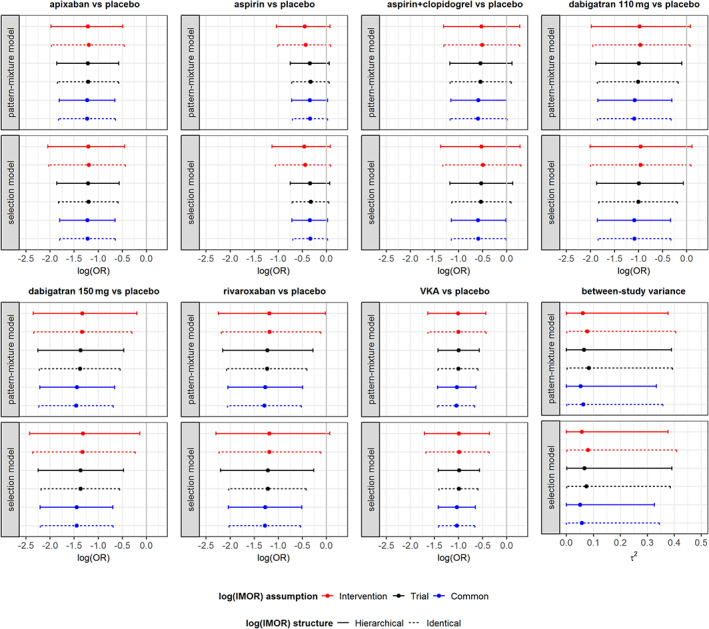
Interval plots on log ORs for basic parameters (posterior mean and 95% credible interval) and between‐trial variance (τ^2^; posterior median and 95% credible interval) when there are moderate and unbalanced missing outcome data (MOD) in the network.^48^ Results are compared in terms of model for MOD (pattern‐mixture, model selection model), structure (hierarchical, identical), and assumption (intervention‐specific, trial‐specific, common‐within‐network) for prior normal distribution on log IMOR assuming missing at random. IMOR, informative missingness odds ratio; OR, odds ratio [Colour figure can be viewed at wileyonlinelibrary.com]

**Figure 3:**
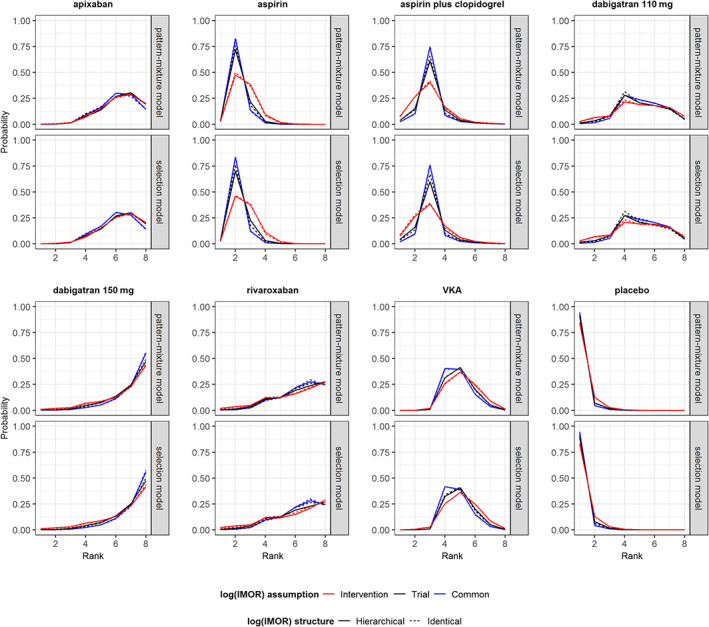
Rankograms of seven interventions when there are moderate and unbalanced missing outcome data (MOD) in the network.^48^ Posterior mean rank probabilities are compared in terms of model for MOD (pattern‐mixture model, selection model), structure (hierarchical, identical) and assumption (intervention‐specific, trial‐specific, common‐within‐network) for prior normal distribution on log IMOR under missing at random. IMOR, informative missingness odds ratio [Colour figure can be viewed at wileyonlinelibrary.com]

Different assumptions about the prior structure of log IMOR appeared to implicate mostly on the width of 95% CrIs for all NMA estimates. Overall, intervention‐specific prior of log IMOR led to wider 95% CrIs for log ORs in both missingness models, whereas common‐within‐network prior led to narrower 95% CrIs for log ORs to some extent. In fact, 95% CrI for log ORs were slightly wider under hierarchical than identical structure. Consequently, the superiority of dabigatran at 110 mg and rivaroxaban against placebo turned into inconclusive when log IMOR was assumed to have intervention‐specific prior structure (Figure 2). Furthermore, *τ*^2^ was relatively lower and slightly more precise under hierarchical structure, especially, for common‐within‐network log IMORs. Since, the common‐within‐network structure provided the narrowest 95% CrIs for log ORs, it led to relatively larger rank probabilities as opposed to intervention‐specific prior structure, especially for aspirin, aspirin plus clopidogrel, and VKA (Figure 3). Results on node‐splitting were in line with those on basic parameters (Supporting Information S.4; Figure S7).

## 4 SIMULATION SETTING

### 4.1 Data generation without missing outcome data

We simulated a triangle network of two‐arm trials and three interventions: placebo, new intervention, and old intervention. The comparison of interest was new versus old intervention. We assumed a typical loop like that in the work of Veroniki et al^49^ with four trials for old intervention versus placebo, three trials for new intervention versus placebo, and one trial for new versus old intervention. To determine the sample size in each arm of every trial, we used information directly from the networks that we collected in our previous empirical work.^27^ For each trial, we considered equally sized arms with sample size generated from a uniform distribution with support in the range defined by the second and third quartile of the arm sizes (Supporting Information S.5; Figure S8(a))

$$n_{i,k}^{E}=n_{i,P}^{C}∼U\left(102,187\right),k=N,O\;\left(\text{placebo‐controlled trials}\right)$$

$$\;n_{i,N}^{E}=n_{i,O}^{C}∼U\left(128,241\right)\;\left(\text{old‐controlled trials}\right),$$

where *N*, *O*, and *P* stand for new intervention, old intervention and placebo, respectively, whereas *E* and *C* stand for experimental and control arm, respectively.

We considered a binary (beneficial) outcome measured in the log OR scale. We assumed *μ*_*NP*_ =  log(2) and *μ*_*OP*_ =  log(1.5) to be the underlying log OR for new and old intervention against placebo, respectively, whereas we obtained the underlying log OR for new versus old intervention through the consistency equation

$$\mu_{\text{NO}}=\mu_{\text{NP}}-\mu_{\text{OP}}+\text{IF},$$

with *IF* being sampled from the t‐distributions *t*(*μ* = 0, *σ*^2^ = 0.44^2^, *df* = 3) and *t*(*μ* = 1, *σ*^2^ = 0.44^2^, *df* = 3) to reflect low and moderate inconsistency on average, respectively, according to our empirical work (Supporting Information S.5; Figure S8(b)).^27^

We generated the number of events in each arm of every trial using the data‐generating model (DGM) described by Hartung and Knapp for a random‐effects pairwise meta‐analysis.^50^, ^51^ The description of this DGM is available as in Supporting Information (S.6). Using information from our network collection,^27^ *initial* event risks for the control arms were generated from a uniform distribution with support in the range defined by the second and third quartile of the event risks (Supporting Information S.5; Figure S8(c))

$$p_{i,P}^{C,0}∼U\left(0.27,0.40\right)\;\text{and}\;p_{i,O}^{C,0}∼U\left(0.63,0.76\right)$$

for placebo‐controlled and old‐controlled trials, respectively.

We incorporated *τ*^2^ (assumed common‐within‐network) in the DGM assuming smaller variability in log odds for placebo (Supporting Information S.5; Figure S8(d)) but equal in log odds for active arms, respectively. In terms of scenarios for *τ*^2^, we selected the predictive log‐normal distributions *L* *N*(−3.95, 1.34^2^) (median: 0.02; IQR: 0.01‐0.04) and *L* *N*(−2.56, 1.74^2^) (median: 0.08; IQR: 0.03‐0.26) to reflect small and substantial *τ*^2^, respectively. These predictive distributions referred to the expected *τ*^2^ in a future meta‐analysis for all‐cause mortality and a generic healthcare setting, respectively.^52^

Finally, we generated the true probability of being best for each intervention by ordering the simulated true log ORs of placebo comparisons as generated from the normal distribution *N*(*μ*_*kP*_, *τ*^2^) with *k* = *N*, *O* and then calculating the number of times each intervention ranked first out of the total simulations.

### 4.2 Data generation while incorporating missing outcome data

Following the motivating examples (Section 3), we focused only on moderate and large MOD as they affected the performance of the modeling strategies to some extent, contrary to low MOD. Note that, under low MOD, we found that all modeling strategies had almost the same performance for log OR, IF and probability of being best but similar performance for *τ*^2^ (results not shown). To ensure balance in MOD between the compared arms, we generated %MOD in the experimental arm, $q_{i,k}^{E}$ with *k* = *N*, *O*, from *U*(0.05, 0.20) and *U*(0.21, 0.40) to indicate moderate and large MOD, respectively (in line with the “five‐and‐twenty rule,” as described in Supporting Information S.1), and we considered $q_{i,P}^{C}=q_{i,k}^{E}$ with *k* = *N*, *O* and $q_{i,O}^{C}=q_{i,N}^{E}$ for the control arms in placebo‐controlled and old‐controlled trials, respectively. In another scenario, to capture the imbalance in MOD between the compared arms, we assumed placebo to have more MOD than the active arms following our empirical study (Supporting Information S.5; Figure S8(e)) and old intervention to have more MOD in the old‐controlled trials.^27^ Details on the generation of unbalanced MOD are available as in Supporting Information (S.7).

Then, we generated the number of MOD in each arm of every trial through the following binomial distributions:

$$\begin{array}{cc} m_{i,k}^{E} & ∼\text{Bin}\left(q_{i,k}^{E},,,n_{i,k}^{E}\right),k=N,O\\ m_{i,k}^{C} & ∼\text{Bin}\left(q_{i,k}^{C},,,n_{i,k}^{C}\right),k=O,P \end{array}$$

for the experimental and control arm, respectively. We used intervention‐specific log IMORs under the pattern‐mixture model to indicate the outcome among the missing participants in each arm of every trial. Specifically, for each trial, we assumed patients randomized in the new or old intervention to be twice more likely to be missing due to the improvement of their outcome as opposed to patients receiving placebo. We considered *σ*^2^ = 1 for the variance of log IMORs. As another scenario, we assumed MAR on average (ie, $\mu_{i,k}^{\varphi }=0$) with *σ*^2^ = 1. Details on the generation of log IMORs are available as in Supporting Information (S.8).

Then, we used the linkage function as described by Turner et al^8^ (equation 7, there) to obtain the probability of events given observed outcomes, $p_{i,k}^{E,\text{obs}}$ and $p_{i,k}^{C,\text{obs}}$ in arm *k* of trial *i* for the experimental and control arm, respectively. The formula to obtain the probability of observed events in each arm is available as in Supporting Information (S.9). Finally, we generated the number of events given the observed outcomes in each arm of every trial as follows:

$$\begin{array}{cc} r_{i,k}^{E,\text{obs}} & ∼\text{Bin}\left(p_{i,k}^{E,\text{obs}},,,n_{i,k}^{E}-m_{i,k}^{E}\right),k=N,O\\ r_{i,k}^{C,\text{obs}} & ∼\text{Bin}\left(p_{i,k}^{C,\text{obs}},,,n_{i,k}^{C}-m_{i,k}^{C}\right),k=O,P \end{array}$$

for the experimental and control arm, respectively. Table 1 summarizes all simulation scenarios considered in the present study.

**Table 1:** Scenarios for the simulation setup

*Number of trials per comparison*	
Typical loop	*NO* = 1, *NP* = 3, *OP* = 4
***Trial size ( *** $n_{i,k}^{E}=n_{i,k}^{C}=n_{i}$ ***in trial* *i**)***	
Placebo‐controlled trials	*n*_*i*_ ∼ *U*(102, 187)
Old‐controlled trials	*n*_*i*_ ∼ *U*(128, 241)
***Initial event rates of control arm in trial* *i***	
Placebo‐controlled trials	$p_{i,P}^{C,0}∼U\left(0.27,0.40\right)$
Old‐controlled trials	$p_{i,O}^{C,0}∼U\left(0.63,0.76\right)$
***Balanced risk of missing outcome data ( *** $q_{i,k}^{E}=q_{i,k}^{C}=q_{i}$ ***in trial**i**)***	
Moderate	*q*_*i*_ ∼ *U*(0.05, 0.20)
Large	*q*_*i*_ ∼ *U*(0.21, 0.40)
***Unbalanced risk of missing outcome data ( *** $q_{i,k}^{E}<q_{i,k}^{C}$ ***in trial**i**)***	
Moderate	$q_{i,k}^{E}∼U\left(0.05,0.10\right)$, $q_{i,k}^{C}∼U\left(0.11,0.20\right)$
Large	$q_{i,k}^{E}∼U\left(0.21,0.30\right)$, $q_{i,k}^{C}∼U\left(0.31,0.40\right)$
***Missingness mechanisms via log (IMOR)***	
Informative	*φ*_*i*,*P*_ ∼ *TN*(*μ* = −log (2), *σ*^2^ = 1, *a* = log (1))
	*φ*_*i*,*k*_ ∼ *TN*(*μ* = log (2), *σ*^2^ = 1, *a* = log (1)) *k* = *N*, *O*
Missing at random	*φ*_*i*,*k*_ ∼ *N*(0, 1) *k* = *N*,*O*,*P*
***Treatment effects***	
Basic parameters	*LOR*_*NP*_ = log (2), *LOR*_*OP*_ = log (1.5)
Functional parameter	*LOR*_*NO*_ = *LOR*_*NP*_ − *LOR*_*OP*_ + *IF*
***Loop inconsistency***	
Inconsistency factor (IF)	*IF* ∼ *t*(*μ* = 0, *σ*^2^ = 0.44^2^, *df* = 3) (low)
	*IF* ∼ *t*(*μ* = 1, *σ*^2^ = 0.44^2^, *df* = 3) (moderate)
***Common between‐trial variance***	
Predictive distribution	*τ*^2^ ∼ *Lν*(−3.95, 1.34^2^) (small)
	*τ*^2^ ∼ *Lν*(−2.56, 1.74^2^) (substantial)
***Probability of being best***	
New intervention	93% and 76% for small and substantial *τ*^2^, respectively
Old intervention	7.3% and 24% for small and substantial *τ*^2^, respectively
Placebo	0% and 0.1% for small and substantial *τ*^2^, respectively

Note: C: control; E: experimental arm; IF: inconsistency factor; IMOR: informative missingness odds ratio; LOR: log odds ratio; N: new intervention; O: old intervention; P: placebo.

Typical loop as defined by Veroniki et al.^49^

Using predictive log‐normal distributions that correspond to all‐cause mortality and generic health setting for small and substantial between‐trial variance, respectively.^52^

### 4.3 Results presentation and model specification

For each scenario, we simulated 1000 triangle networks and, for each scenario, we evaluated the posterior distribution of *μ*_*NO*_, *τ*^2^, IF and probability of being best for each intervention. For each NMA estimate, we used interval plots to present the simulation results in order to fully reflect the dispersion of the results for each scenario. We decided to present in the main text only results on prior structures of log IMOR under pattern‐mixture model as it is the most frequently reported model in systematic reviews.^19^ Results on prior structures of log IMOR under selection model are available in Supporting Information (S.11; Figures S10‐S13). Furthermore, we focused on informative MOD with moderate and large extent for being the most plausible scenarios in a medical setting. Results on prior structures of log IMOR when MOD are MAR can be found in Supporting Information (S.12; Figures S14‐S17). Simulations and analyses were performed in the line with the motivating examples (Section 3). For each of the 1000 simulations, thinning equal to 3 was used for 20 000 updates and a burn‐in of 2000 MCMC samples.^38^

## 5 RESULTS

### 5.1 Posterior distribution of log OR (**μ**_***NO***_)

**Figure 4:**
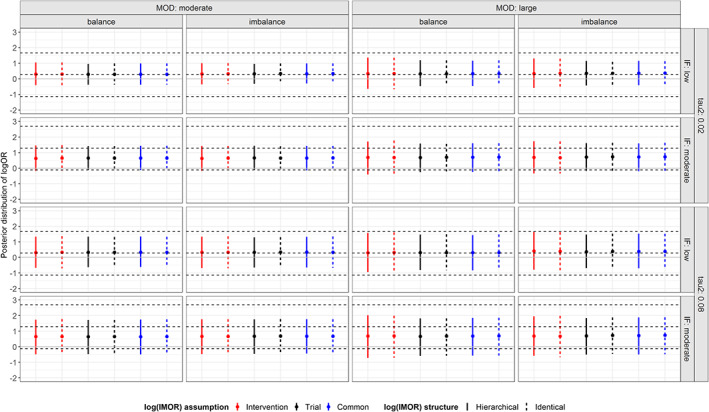
Posterior distribution of log OR (between new and old intervention) under informative missingness while using pattern‐mixture model and accounting for the extent of missing outcome data (moderate, large), balance of missing outcome data (balance, imbalance), extent of τ^2^ (small, substantial), and extent of inconsistency (low, moderate). The horizontal dotted lines reflect the 95% interval and mean of the simulated distribution of log OR under low and moderate true inconsistency. IF, inconsistency factor; MOD, missing outcome data [Colour figure can be viewed at wileyonlinelibrary.com]

Under low inconsistency, the posterior mean of log OR almost converged with the simulated distribution for all prior structures of log IMOR regardless of extent and balance of MOD (Figure 4). Credible intervals were broadly similar for moderate MOD. Subtle differences in the CrIs were observed for large MOD: assuming intervention‐specific log IMORs led to slightly wider CrIs (similarly for identical and hierarchical structure) compared to trial‐specific and common‐within‐network prior structure. Substantial *τ*^2^ naturally led to wider CrIs compared to small *τ*^2^ without affecting the point estimate. With moderate inconsistency, the posterior distribution of log ORs deviated from the simulated distribution in all prior structures of log IMOR.

### 5.2 Posterior distribution of **τ**^2^

**Figure 5:**
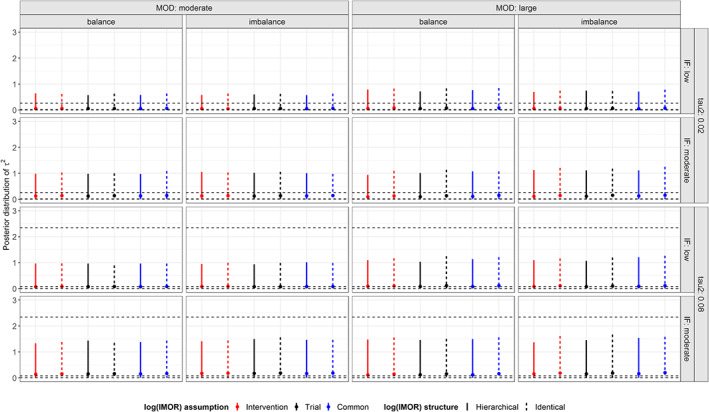
Posterior distribution of τ^2^ under informative missingness while using pattern‐mixture model and accounting for the extent of missing outcome data (moderate, large), balance of missing outcome data (balance, imbalance), extent of τ^2^ (small, substantial), and extent of inconsistency (low, moderate). The horizontal dotted lines reflect the 95% interval and median of the simulated distribution of small and substantial τ^2^. IF, inconsistency factor; MOD, missing outcome data [Colour figure can be viewed at wileyonlinelibrary.com]

Posterior median of *τ*^2^ was close to zero in all prior structures of log IMOR for low inconsistency and small *τ*^2^, whereas, as expected, it increased for moderate inconsistency and/or substantial *τ*^2^. For moderate MOD and low inconsistency, posterior median and CrI for *τ*^2^ were quite similar across all prior structures of log IMOR, whereas for large MOD, posterior median for *τ*^2^ increased slightly with wider widths of CrIs that slightly differed for different assumptions of log IMOR within the hierarchical and identical structure (Figure 5). Identical structure led systematically to slightly wider CrIs in most prior structures of log IMOR as compared to hierarchical structure. In addition, the point estimates were slightly larger for identical structure, particularly, for moderate inconsistency and large MOD.

### 5.3 Posterior distribution of IF

**Figure 6:**
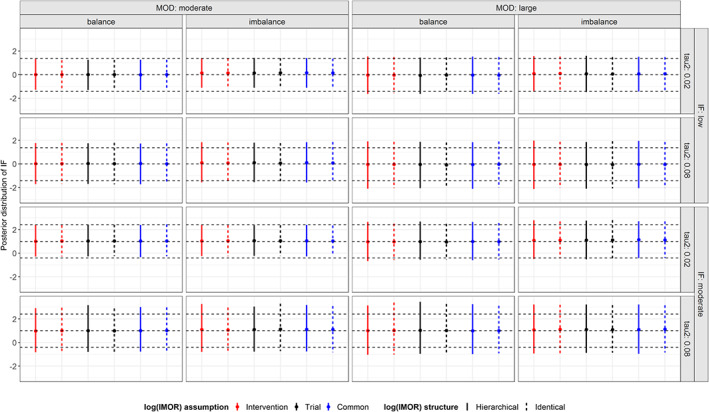
Posterior distribution of inconsistency factor (IF) under informative missingness while using pattern‐mixture model and accounting for the extent of missing outcome data (moderate, large), balance of missing outcome data (balance, imbalance), extent of τ^2^ (small, substantial) and extent of inconsistency (low, moderate). The horizontal dotted lines reflect the 95% interval and mean of the simulated distribution of low and moderate IF. MOD, missing outcome data [Colour figure can be viewed at wileyonlinelibrary.com]

Under low inconsistency, the posterior mean of IF was almost zero (ie, evidence of consistency on average) in all prior structures of log IMOR and for all scenarios (Figure 6). Overall, CrIs were similarly wider in the presence of substantial *τ*^2^. In the presence of moderate inconsistency, all prior structures of IMOR estimated the true IF, and hence, the point estimates deviated from zero irrespective of extent and balance of MOD.

### 5.4 Posterior distribution of probability of being best

The posterior mean of the probability of being best for new intervention was consistently below the simulated distribution for all prior structures of log IMOR, especially, for large MOD and low inconsistency (Supporting Information S.10; Figure S9). Interestingly, contrary to low inconsistency, moderate inconsistency lowered the posterior mean of the probability of being best the least for all prior structures. Within each scenario, the posterior mean of the probability of being best was similar across all prior structures but slightly larger for unbalanced MOD. Nevertheless, intervention‐specific log IMORs led to slightly smaller posterior mean of the probability of being best, especially, for large MOD, moderate inconsistency and small *τ*^2^. The posterior mean of the probability of being best almost overlapped with the simulated distribution for moderate MOD, small *τ*^2^, and moderate inconsistency. Results on the posterior mean of the probability of being best for old intervention and placebo can be found in the Supporting Information (results not shown). Overall, different scenarios and prior structures of log IMOR did not impact on the hierarchy of the interventions.

## 6 DISCUSSION

Using three published networks with different extent of MOD as motivating examples, we compared pattern‐mixture with selection model while considering six different prior structures of log IMOR that reflected our prior beliefs about the (dis)similarity of log IMORs within the network. Then, on the basis of the results from the motivating examples, we set up a simulation study using empirical‐based scenarios to evaluate more in‐depth the performance of these prior structures of log IMOR in terms of posterior distribution of log OR, *τ*^2^, IF and probability of being best per intervention. We focused on the performance of prior structures when informative MOD (the most plausible scenario in a medical setting) were analyzed under MAR (the recommended primary analysis for MOD). To our knowledge, this is the first simulation study that evaluates statistical modeling of aggregated MOD using Bayesian approaches.

Ultimate goal of the present study was to supplement our observations from our empirical study on these modeling strategies.^27^ In our empirical study,^27^ we used Bland‐Altman plots to investigate the degree of agreement among these strategies in terms of NMA estimates. The majority of the networks considered had either low or moderate and balanced MOD. Therefore, we were not able to conclude on the agreement of the strategies when MOD were large or moderate and unbalanced. Furthermore, with an empirical study, we cannot infer on performance measures, such as bias. Consequently, the present simulation study addressed the aforementioned limitations and, additionally, allowed us investigating the performance of the strategies under different scenarios for the NMA estimates in order to understand the circumstances that may compromise the performance of the strategies.

The last two motivating examples agreed with our empirical study,^27^ which indicated that, for moderate and large MOD, (hierarchical and identical) intervention‐specific prior structure of log IMOR led to larger posterior standard deviation of log ORs as compared to trial‐specific and common‐within‐network prior structures—the latter two led overall to similar posterior distributions of log ORs. White et al also noticed that the uncertainty around meta‐analysis log OR was larger for intervention‐specific prior structure while similar for trial‐specific and common‐within‐network prior structures.^20^ Our simulation revealed this pattern for large MOD only, regardless of balance of MOD. This performance of intervention‐specific prior structure was anticipated as it assumes MOD to be differently informative in different interventions, and therefore, it substantially down‐weights trials with moderate or large MOD leading to larger posterior standard deviation of summary log OR.^33^

Furthermore, both the present study and our empirical study^27^ demonstrated that hierarchical prior structure of log IMOR led to slightly more precise *τ*^2^ compared to identical prior structure, particularly for moderate, unbalanced MOD (Section 3.3). According to our simulation study, this performance was more profound for large MOD with concurrence of inconsistent evidence and/or substantial *τ*^2^. The extent of informative missingness (as quantified via log IMOR) was simulated to vary across the included trials for the same intervention (Equation (1) in Supporting Information S.8); however, the identical structure did not capture this variability yielding spuriously narrower CrIs for the study‐specific log ORs as compared to hierarchical structure which, in turn, led to relatively larger *τ*^2^ and uncertainty thereof.

The third motivating example indicated that common‐within‐network prior structure provided slightly more precise estimation of *τ*^2^ compared to intervention‐ and trial‐specific prior structures. Nevertheless, in the simulation study, this pattern was less obvious for moderate, unbalanced MOD, and small *τ*^2^. Possible explanation may be that the motivating example had almost three times more trials than the simulated networks, and in conjunction with the common‐within‐network being the least data demanding structure of log IMOR, *τ*^2^ was estimated with relatively more precision for this prior structure in the motivating example.

We found that pattern‐mixture and selection models gave almost identical results for each prior structure of log IMOR in the motivating examples and simulation study (Supporting Information S.12). While these two models lead to fundamentally opposite factorizations of the joint distribution of the missingness indicator and outcome, the parameter of interest *p*_*i*,*k*_ is not affected by this factorization, because, in both models, *p*_*i*,*k*_ is function of *q*_*i*,*k*_ and $\varphi_{i,k}^{l}$ (see Equations (2) and (3)) with the same informative prior distribution being assigned on $\varphi_{i,k}^{l}$. Where these models differ is on the conditional probabilities that define $\varphi_{i,k}^{l}$ (Section 2.2.3). Nevertheless, if one is interested in investigating the interventions to subgroups of trials that are believed to have different measurement patterns, then pattern‐mixture model may be the proper option.^18^ For example, (as judged, for instance, by the Cochrane's risk of bias tool; chapter 8 in the work of Higgins and Green^6^), if poorly conducted trials have more MOD than well‐conducted trials—and the researcher believes that compared to those leaving poorly conducted trials, patients completing these trials may be more likely to have experienced the beneficial outcome—the researcher should investigate whether the pattern of outcomes in these two trial settings may affect differently the interventions compared. To our knowledge, pattern‐mixture model has not been applied yet in series of trials with the aim to provide further insights on the effectiveness of the interventions on subgroups of different patterns of outcome. Instead, if one is interested in the effectiveness of the interventions in the whole population, then pattern‐mixture and selection models may be used interchangeably in the analysis of series of trials—although, in principle, the latter is a more natural option^18^ as it directly reflects the taxonomy of missingness mechanisms as described by Little and Rubin.^15^

Deciding on the assumption for log IMOR shall be primarily tailored to empirical knowledge about the intervention and trial characteristics for the condition under investigation.^8^, ^20^ For example, contrary to active‐controlled trials in schizophrenia, placebo‐controlled trials lead to greater dropout rate among patients without improvement in their outcomes.^53^, ^54^ Then, the researcher can consider placebo‐ and active‐specific priors on log IMOR and further investigate the sensitivity of results to using identical and hierarchical structures. In another example, multi‐center trials in psychiatry tend to have higher dropout rate (and hence log IMOR in these trials is more likely to be different from 0) than single‐center trials; if log IMORs are believed not to differ among the compared interventions, and the researcher has collected for each trial information on the number of centers, then he/she should assign hierarchical, multi‐center‐specific, and single‐center‐specific priors on log IMOR so that log IMORs are different yet related in the corresponding trials. In our simulation study, the proper prior structure of log IMOR was intervention‐specific because we assumed placebo to trigger different missingness mechanisms as opposed to new and old intervention. However, by misspecifying the prior structure using trial‐specific or common‐within‐network prior structure appeared to affect the uncertainty around the log OR leading to narrower CrIs of log OR when MOD were moderate or large. While the inferences about the relative effectiveness of the interventions were not be affected in our simulations, the robustness of the inferences for dabigatran 110 mg and rivaroxaban against placebo (third motivating example) were sensible to the prior structure of log IMOR.

In the present study, we addressed aggregated MOD using two popular models for MOD and six different prior structures of log IMOR without accounting, in addition, for important effect modifiers. van Buuren et al^55^ developed a multiple imputation (MI) model that incorporates a delta parameter like IMOR under pattern‐mixture model to investigate the degree of departure from MAR in survival analysis in a clinical trial. Extending this model to operate in a collection of trials investigating two or more interventions is an interesting yet unexplored area (to our knowledge) for further work. Provided that we had access to individual patient data (IPD) and enough studies in the network to allow for effect‐modification adjustments, MI based on missing not at random (MNAR) assumptions would be a more elegant modeling strategy—though computationally more intensive. This is because MI is already increasingly used for offering a relatively simply and attractive way to account also for the uncertainty induced by imputations (commonly applied under MAR) while adjusting the model for important predictors. In addition, IPD has been often considered as gold standard for synthesizing series of trials as it allows a more rigorous investigation of statistical heterogeneity that—contrary to standard aggregated analysis—protects against the risk for ecological bias, particularly for subject‐level characteristics.^35^ Since addressing MOD is based on untestable assumptions about missing outcomes (the popular MAR assumption cannot be tested from the observed outcomes), extending “standard” MI to investigate the sensitivity to MAR via MNAR models offers more flexibility.

The limitations of our study pertain mostly to the simulation setup. Firstly, we used Bayesian approaches as we intended to compare different Bayesian modeling strategies for binary MOD in terms of NMA estimates. Consequently, we preferred not to infer on the performance of the models in terms of frequentist measures, such as type I error, efficiency, and coverage; contrariwise, our inferences stemmed from the posterior distribution of the NMA estimates for different scenarios and models. Secondly, we considered a simple network of three interventions and two‐arm trials with binary outcome data (the most prevalent outcome type in systematic reviews^23^). A more complex network with the addition of multi‐arm trials—a “typical” network in practice^24^—will shed more light on the implications of network complexity on the NMA estimates across different prior structures of log IMOR. For instance, in a complex yet sparse network (where the number of trials and observed comparisons are limited), identical prior structure may perform better to hierarchical structure as it is the least data demanding (alike the common‐within‐network prior structure). Thirdly, we did not investigate the impact of event frequency since we considered only frequent events. As noted in the work of Carpenter and Kenward,^56^ “if an event (eg, death or a serious side effect) is rare, missing [outcome] data on very few patients can markedly alter estimated event rates,” and therefore, affect substantially the NMA estimates. Fourthly, the degree of unbalanced MOD considered in the simulation setup was much smaller than the total extent of MOD in each trial (Supporting Information S.7). Consequently, the width of CrI for log OR under common‐within‐network and trial‐specific prior structures (they assume MOD to be equally informative in the whole network and within each trial, respectively, and hence, they down‐weight trials with unbalanced MOD in the compared arms^33^) remained narrower than the width of CrI for log OR under intervention‐specific prior structure when MOD were unbalanced. A much larger imbalance of MOD may have resulted in more imprecise log OR under common‐within‐network and trial‐specific prior structures. However, we did not observe such extent of imbalance in our empirical study (Supporting Information S.5; Figure S8(f)). Lastly, we dealt with convergence issues (via inspection of the trace and autocorrelation plots) after applying identical common‐within‐network in both pattern‐mixture and selection models; this issue was not tackled after we increased thinning at 6 and 10 (Figures not shown).

**Recommendations for the reviewer**

- The reviewer should decide in advance on the proper prior structure of log IMOR to address aggregated MOD that best aligns with the condition investigated and the interventions forming the network; otherwise, misspecification of the prior structure may lead to spurious estimation of the uncertainty around log OR with implications for the conclusions—as shown in the motivating examples and simulation study.
- Pattern‐mixture and selection models can be applied interchangeably to infer on the effectiveness of the compared interventions on the whole population.
- Both identical and hierarchical structure may be considered in the context of a sensitivity analysis; though, we expect log IMORs to be different (since the extent of MOD will differ across trial‐arms, among other reasons) yet related to each other, and hence, we regard hierarchical structure to be more plausible in practice.

## 7 CONCLUSIONS

Assuming MAR on average as a starting point to analyze informative MOD under different prior structures of log IMOR appeared to implicate mainly the precision of the NMA estimates without affecting our conclusions about the effectiveness and the hierarchy of the interventions. Nevertheless, the inferences from the present simulation study were greatly restricted by the scenarios considered. Reviewers should decide already at the protocol of the systematic review on the prior structure of log IMOR according to the condition and interventions investigated. Our results may be also generalized to conventional meta‐analyses with binary outcome.

## Supporting information

SIM_8207‐Supp‐0001‐Supplementary Information.docx
