# Supplementary material for: Participants' outcomes gone missing within a network of interventions: Bayesian modeling strategies
Source: Stat Med. 2019 May 27;38(20):3861–79. doi: 10.1002/sim.8207 (PMC7754380; doi:10.1002/sim.8207)
Supplement: Supplementary file 1 — SIM_8207‐Supp‐0001‐Supplementary Information.docx [file SIM-38-3861-s001.docx]

**Supplementary material**

1. **Classification of networks based on the amount of missing outcome data**

To classify an NMA as having low, moderate or large missing outcome data (MOD), we developed a decision rule that compared the median of total percentage MOD (%MOD) across the included trials with the ‘five-and-twenty rule’ as proposed by Sackett et al.^1^ Therefore, we considered MOD to be low for median up to 5%, large for median above 20% and moderate otherwise. ^2^

To decide whether an NMA included trials with balance or unbalanced MOD, we compared the median of the difference in %MOD between the compared arms across the included trials with the ‘threshold of balance’, 6.5%. ^2^ This threshold was obtained as the intersection of the densities formed by trials with and without balanced MOD in the compared arms as determined using a two-sided Pearson's chi-squared test statistic and significance level 5% to test the null hypothesis that the compared arms have the same %MOD. ^2^ Networks with a median difference in %MOD between the compared arms larger than 6.5% were considered to have trials with unbalanced MOD.

Reference

1. Sackett DL, Richardson WS, Rosenberg WM, Haynes RB. Evidence-Based Medicine: How to Practice and Teach EBM. New York, Churchill Livingstone; 1997.
2. Spineli LM. An empirical comparison of Bayesian modelling strategies for missing binary outcome data in network meta-analysis. BMC Med Res Methodol. 2019. In press. DOI: 10.1186/s12874-019-0731-y.
3. **Motivating example:** **low missing outcome data (Example 1)**

**
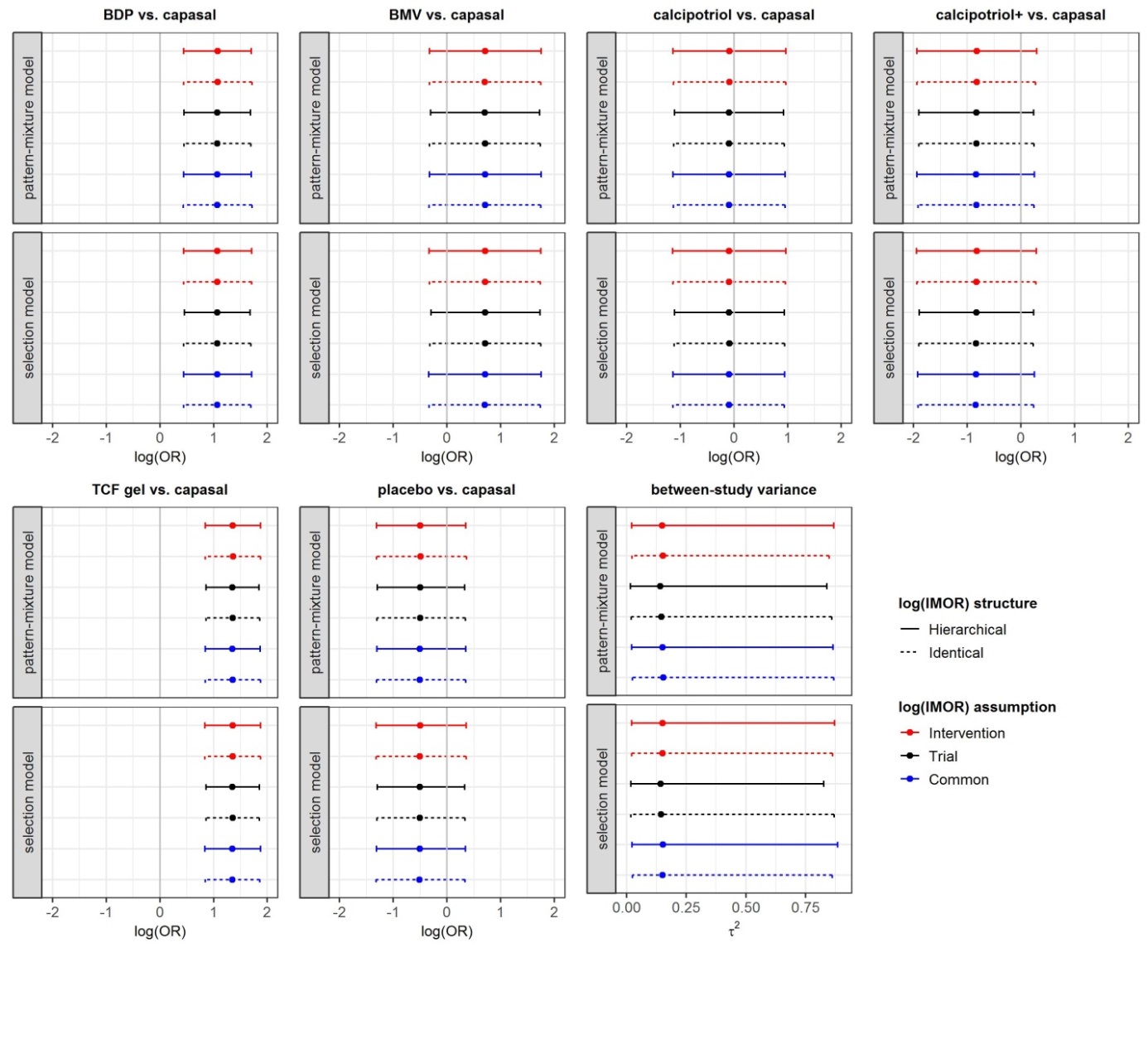
**

**Figure S1.** Intervals plots on log ORs for basic parameters (posterior mean and 95% credible interval) and between-trial variance ($\tau^{2}$; posterior median and 95% credible interval) when there are low missing outcome data (MOD) in the network.^1^ Results are compared in terms of model for MOD (pattern-mixture model, selection model), structure (hierarchical, identical) and assumption (intervention-specific, trial-specific, common-within-network) for prior normal distribution on log IMOR under missing at random. OR, odds ratio; IMOR, informative missingness odds ratio.

1. Bottomley JM, Taylor RS, Ryttov J. The effectiveness of two-compound formulation calcipotriol and betamethasone dipropionate gel in the treatment of moderately severe scalp psoriasis: a systematic review of direct and indirect evidence. Curr Med Res Opin. 2011;27(1):251-268.


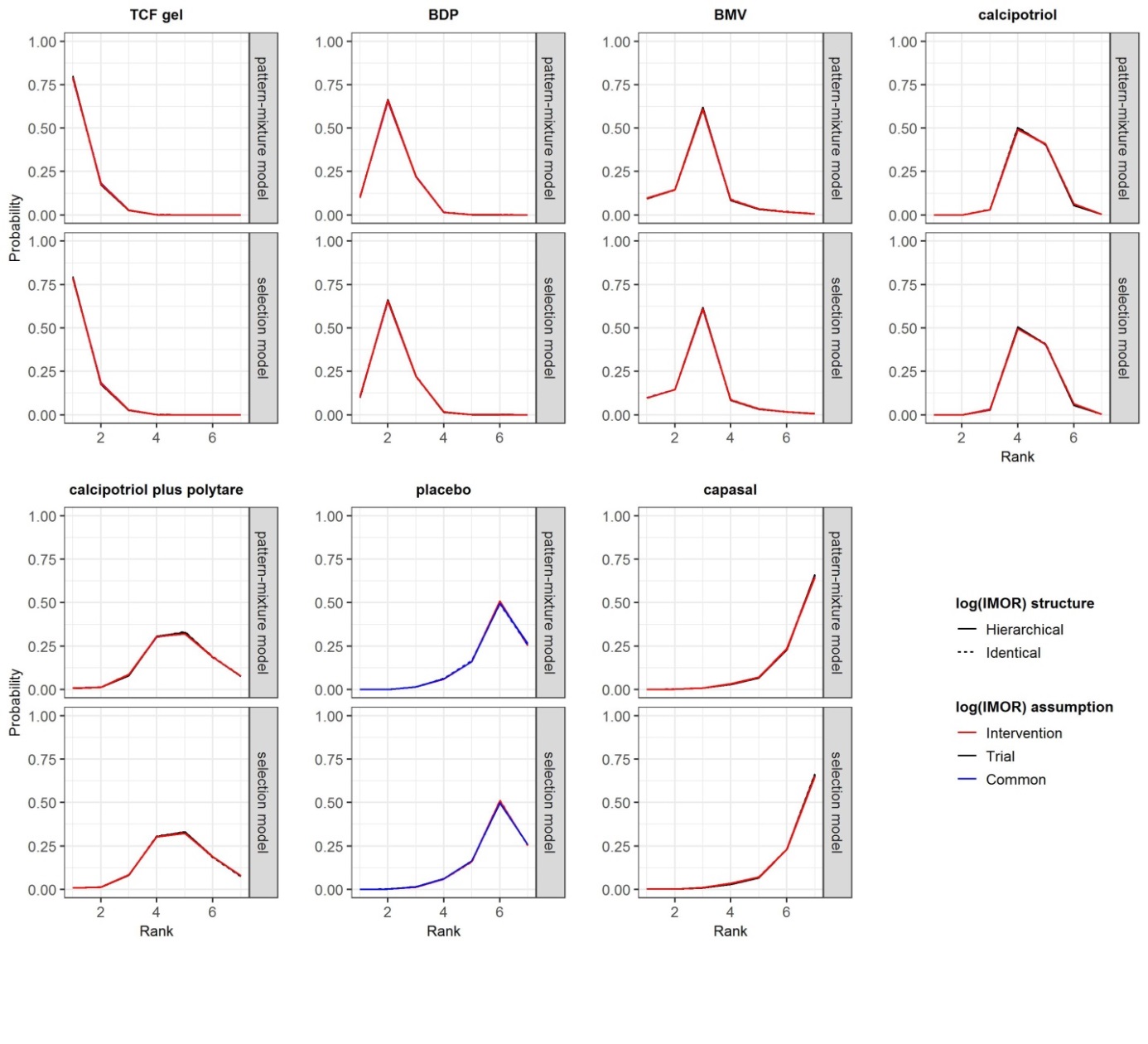


**Figure S2.** Rankograms of seven interventions when there are low missing outcome data (MOD) in the network.^1^ Posterior mean rank probabilities are compared in terms of model for MOD (pattern-mixture model, selection model), structure (hierarchical, identical) and assumption (intervention-specific, trial-specific, common-within-network) for prior normal distribution on log IMOR under missing at random. IMOR, informative missingness odds ratio.

1. Bottomley JM, Taylor RS, Ryttov J. The effectiveness of two-compound formulation calcipotriol and betamethasone dipropionate gel in the treatment of moderately severe scalp psoriasis: a systematic review of direct and indirect evidence. Curr Med Res Opin. 2011;27(1):251-268.


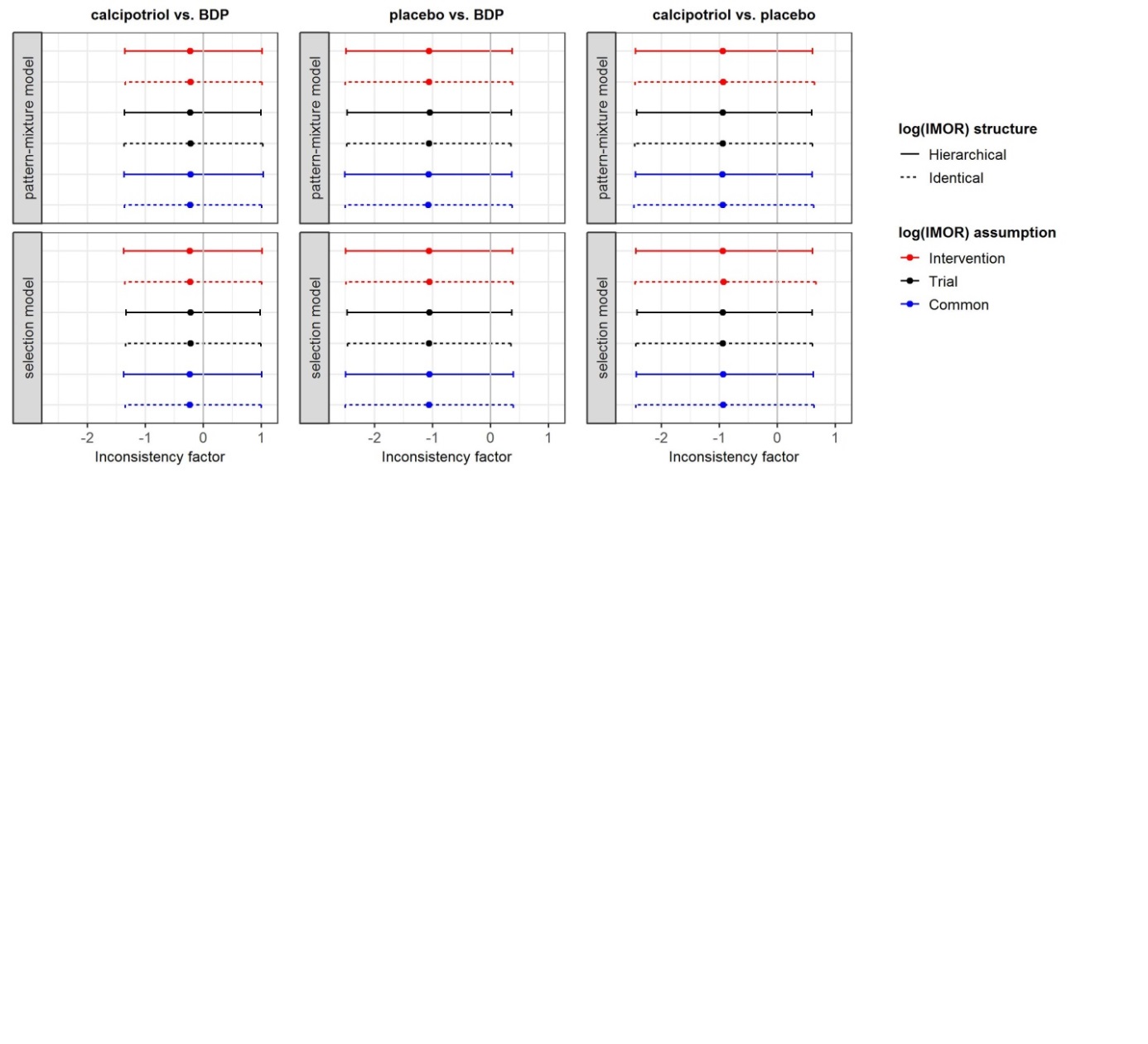


**Figure S3.** Interval plots on inconsistency factor (posterior mean and 95% credible interval) according to node-splitting approach when there are low missing outcome data (MOD) in the network.^1^ Results are compared in terms of model for MOD (pattern-mixture model, selection model), structure (hierarchical, identical) and assumption (intervention-specific, trial-specific, common-within-network) for prior normal distribution on log IMOR under missing at random. IMOR, informative missingness odds ratio.

1. Bottomley JM, Taylor RS, Ryttov J. The effectiveness of two-compound formulation calcipotriol and betamethasone dipropionate gel in the treatment of moderately severe scalp psoriasis: a systematic review of direct and indirect evidence. Curr Med Res Opin. 2011;27(1):251-268.
2. **Motivating example:** **moderate and balanced missing outcome data (Example 2)**


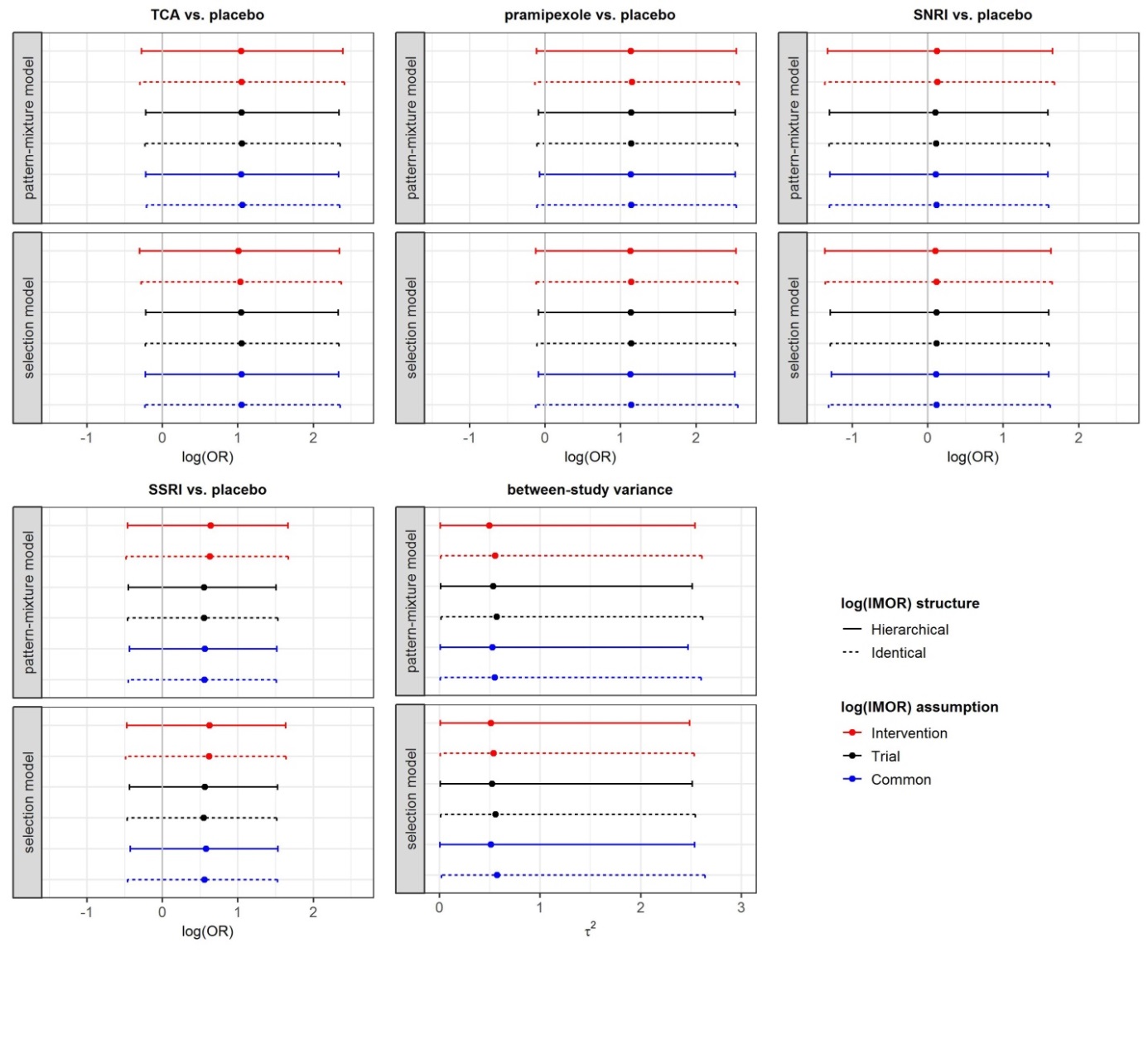


**Figure S4.** Interval plots on log ORs for basic parameters (posterior mean and 95% credible interval) and between-trial variance ($\tau^{2}$; posterior median and 95% credible interval) when there are moderate and balanced missing outcome data (MOD) in the network.^1^ Results are compared in terms of model for MOD (pattern-mixture model, selection model), structure (hierarchical, identical) and assumption (intervention-specific, trial-specific, common-within-network) for prior normal distribution on log IMOR under missing at random. OR, odds ratio; IMOR, informative missingness odds ratio.

1. Liu J, Dong J, Wang L, et al. Comparative Efficacy and Acceptability of Antidepressants in Parkinson’s Disease: a Network Meta-Analysis. PLoS One. 2013;8(10):e76651.


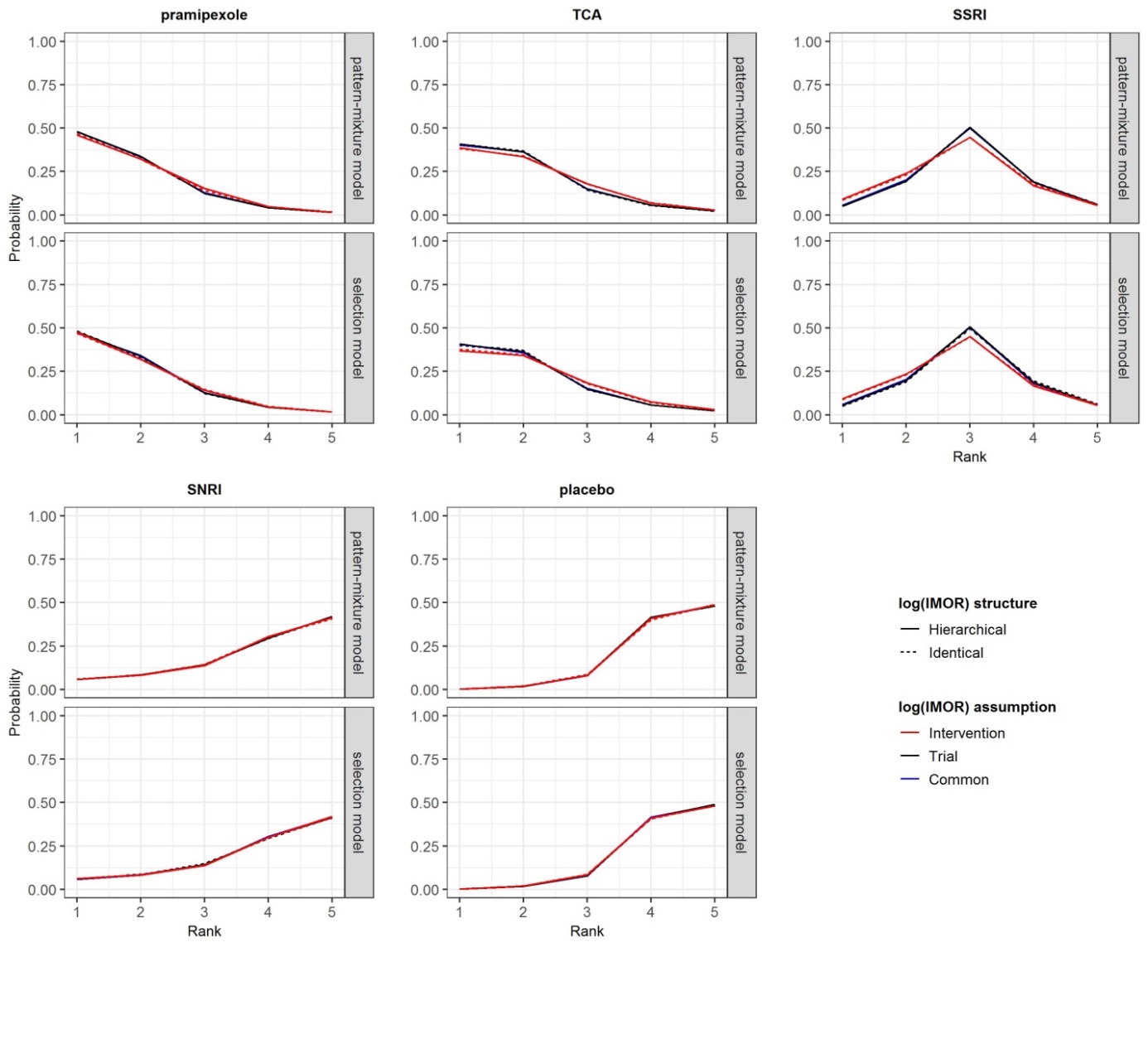


**Figure S5.** Rankograms of five interventions when there are moderate and balanced missing outcome data (MOD) in the network.^1^ Posterior mean rank probabilities are compared in terms of model for MOD (pattern-mixture model, selection model), structure (hierarchical, identical) and assumption (intervention-specific, trial-specific, common-within-network) for prior normal distribution on log IMOR under missing at random. IMOR, informative missingness odds ratio.

1. Liu J, Dong J, Wang L, et al. Comparative Efficacy and Acceptability of Antidepressants in Parkinson’s Disease: a Network Meta-Analysis. PLoS One. 2013;8(10):e76651.


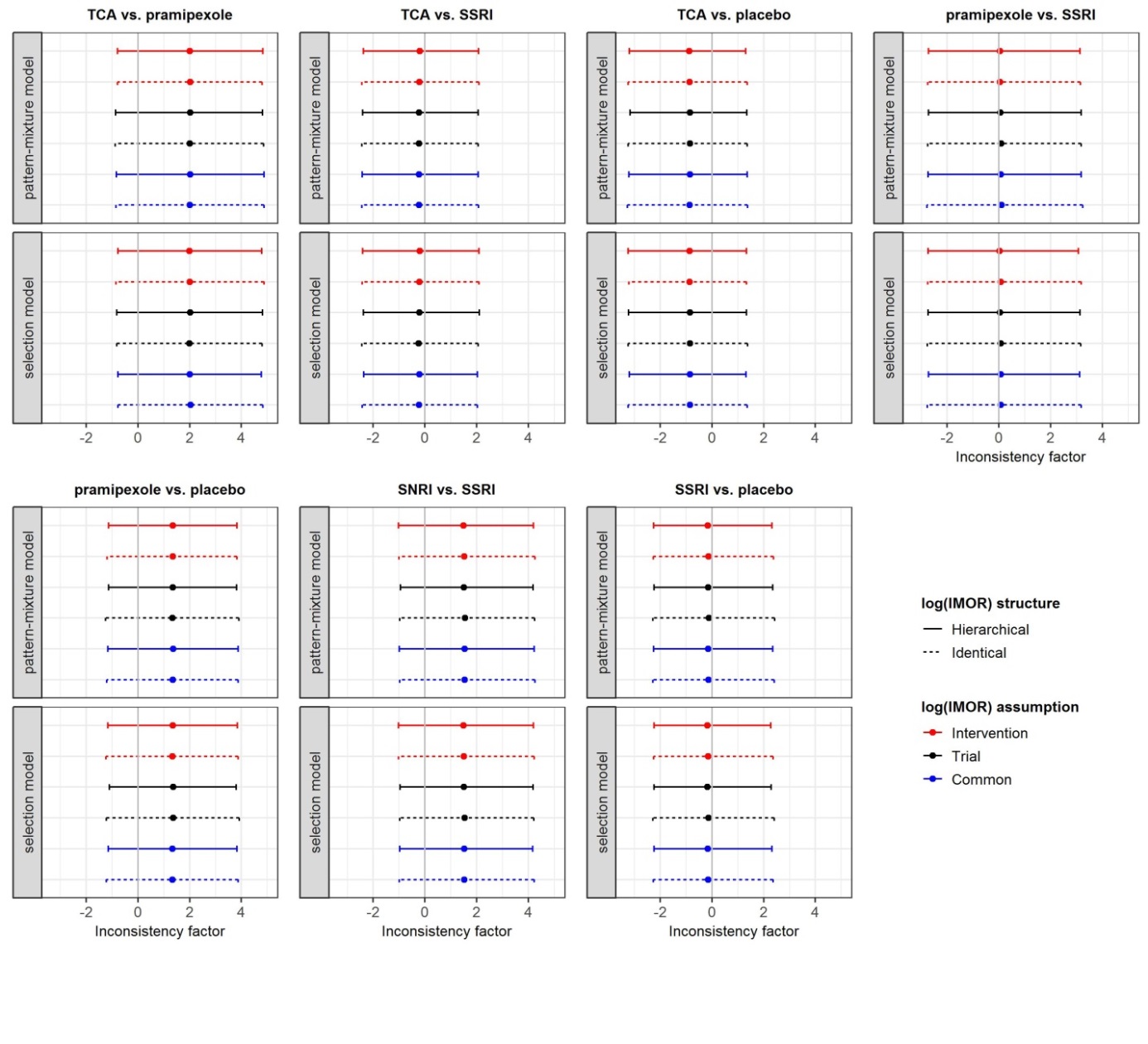


**Figure S6.** Interval plots on inconsistency factor (posterior mean and 95% credible interval) according to node-splitting approach when there are moderate and balanced missing outcome data (MOD) in the network.^1^ Results are compared in terms of model for MOD (pattern-mixture model, selection model), structure (hierarchical, identical) and assumption (intervention-specific, trial-specific, common-within-network) for prior normal distribution on log IMOR under missing at random. IMOR, informative missingness odds ratio.

1. Liu J, Dong J, Wang L, et al. Comparative Efficacy and Acceptability of Antidepressants in Parkinson’s Disease: a Network Meta-Analysis. PLoS One. 2013;8(10):e76651.
2. **Motivating example:** **moderate and unbalanced missing outcome data (Example 3)**


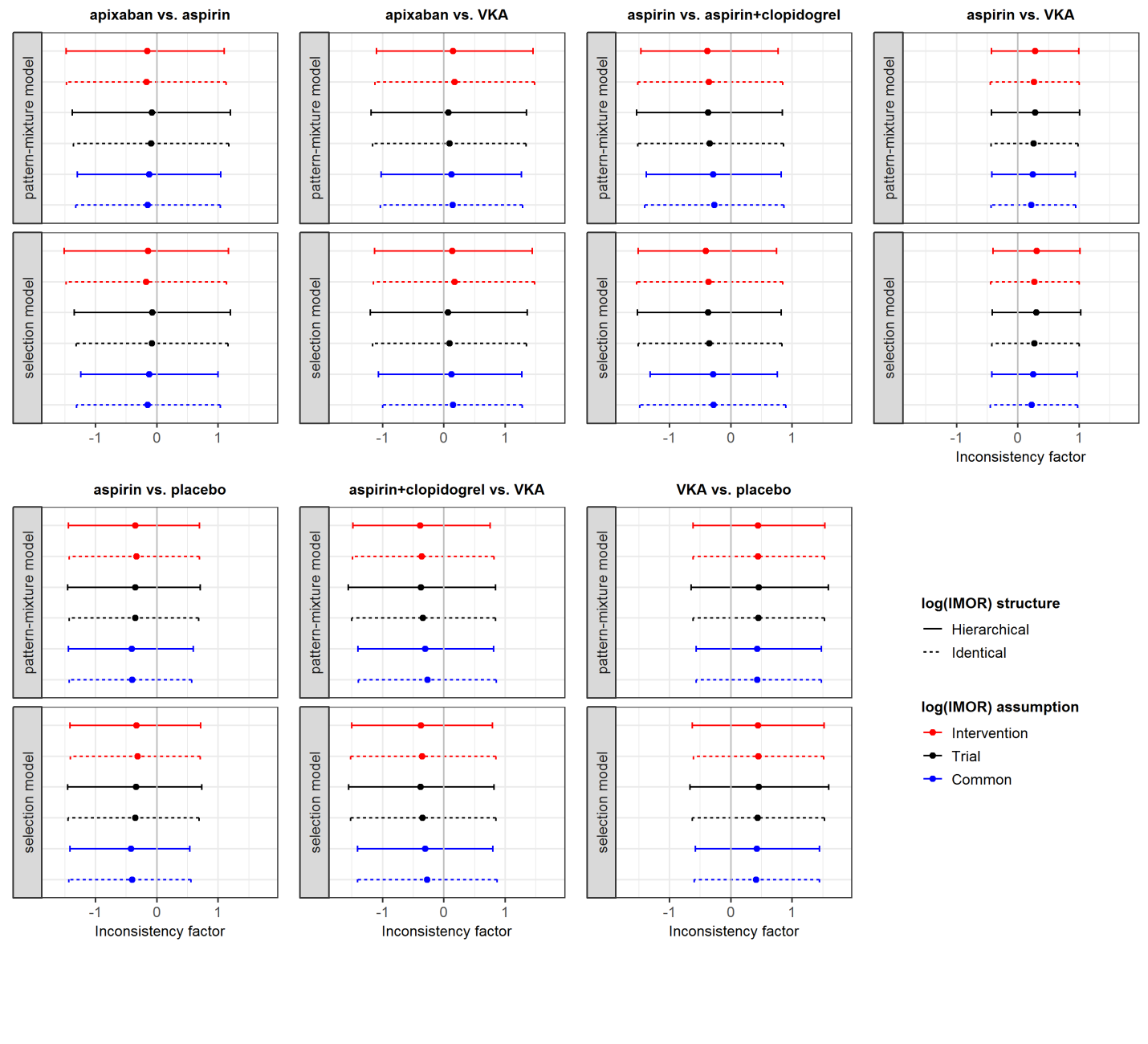


**Figure S7.** Interval plots on inconsistency factor (posterior mean and 95% credible interval) according to node-splitting approach when there are moderate and unbalanced missing outcome data (MOD) in the network.^1^ Results are compared in terms of model for MOD (pattern-mixture model, selection model), structure (hierarchical, identical) and assumption (intervention-specific, trial-specific, common-within-network) for prior normal distribution on log IMOR under missing at random. IMOR, informative missingness odds ratio.

1. Dogliotti A, Paolasso E, Giugliano RP. Current and new oral antithrombotics in non- valvular atrial fi brillation: a network meta-analysis of 79 808 patients. Heart. 2014;100(5):396-405.
2. **Use of empirical evidence to inform simulation scenarios**


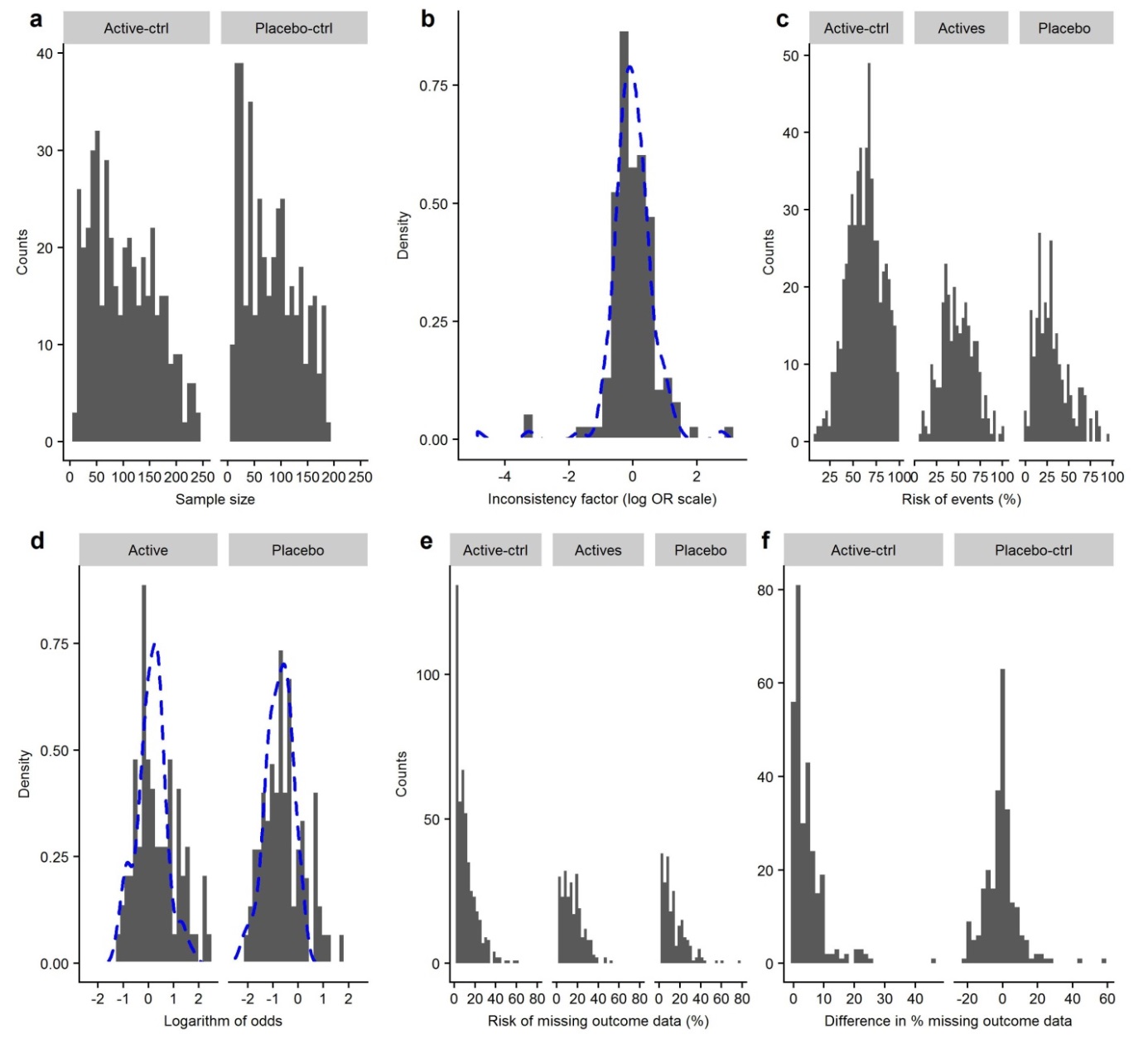


**Figure S8**. A panel of histograms on the distribution of (a) trial-arm sample size across active- and placebo-controlled trials; (b) mean inconsistency factor obtained using identical, intervention-specific prior normal distribution on log IMORs with mean 0 and variance 1 – the fitted density is a t-distribution with mean -0.02, variance 0.44^2^ and 3 degrees of freedom; (c) risk of events in active-controlled trials (first histogram), risk of events in active arms of placebo-controlled trials (second histogram) and risk of events in placebo arms (third histogram); (d) odds in logarithmic scale for active and placebo arm in two-arm placebo-controlled trials – the fitted densities are normal distributions with mean 0.44 and variance 0.97^2^ for active arms, whereas mean -0.59 and variance 0.81^2^ for placebo; (e) risk of missing outcome data in active interventions of active- and placebo-controlled trials (first and second histogram, respectively) and risk of missing outcome data in placebo (third histogram) and (f) difference in percentage missing outcome data between the compared arms across active- and placebo-controlled trials. Ctrl, controlled; OR, odds ratio.

1. **Description of the Hartung and Knapp** **data-generating model**

According to data-generating model by Hartung and Knapp, ^1^ *initial* event risks were generated for the experimental arm $p_{i,k}^{E,0}$ with $k=N,O$ as a function of the selected *initial* event risks in the control arm $p_{i,k}^{C,0}$ with $k=O,P$ and underlying $\mu_{kl}$ ($k=N,O,$ $l=O,P$ and $k\neq l$) through the following equations:

$$p_{i,k}^{E,0}=\frac{p_{i,P}^{C,0}\cdot exp\left( \mu_{kP} \right)}{1-p_{i,P}^{C,0}+p_{i,P}^{C,0}\cdot exp\left( \mu_{kP} \right)}, k=N,O$$

$$p_{i,N}^{E,0}=\frac{p_{i,O}^{C,0}\cdot exp\left( \mu_{NO} \right)}{1-p_{i,O}^{C,0}+p_{i,O}^{C,0}\cdot exp\left( \mu_{NO} \right)}$$

for placebo- and old-controlled trials, respectively. Using information from our network collection, ^2^ we assumed *initial* event risks for the control arms generated from a uniform distribution with support in the range defined by the second and third quartile of the event risks (Figure S8 (c)):

$p_{i,P}^{C,0}\sim U\left( 0.27, 0.40 \right)$ and $p_{i,O}^{C,0}\sim U\left( 0.63, 0.76 \right)$

for placebo- and old-controlled trials, respectively. Then, we calculated the underlying log odds for each arm in every trial as follows:

$${logit}_{i,k}^{E,0}=log\left( \frac{p_{i,k}^{E,0}}{1-p_{i,k}^{E,0}} \right), k=N,O$$

$${logit}_{i,k}^{C,0}=log\left( \frac{p_{i,k}^{C,0}}{1-p_{i,k}^{C,0}} \right), k=O,P$$

for the experimental and control arm, respectively.

Furthermore, we incorporated $\tau^{2}$ (assumed common-within-network) in either arm in order to generate log odds for each arm in every trial via the following normal distributions:

Placebo-controlled

${logit}_{i,k}^{E}\sim N\left( {logit}_{i,k}^{E,0},\frac{{2\tau}^{2}}{3} \right), k=N,O$and${logit}_{i,k}^{C}\sim N\left( {logit}_{i,k}^{C,0},\frac{\tau^{2}}{3} \right), k=O,P$

Old-controlled

$${logit}_{i,N}^{C}\sim N\left( {logit}_{i,k}^{C,0},\frac{\tau^{2}}{2} \right) \mathrm{and} {logit}_{i,O}^{C}\sim N\left( {logit}_{i,k}^{C,0},\frac{\tau^{2}}{2} \right),$$

where, in line with our empirical study, ^2^ we assumed smaller variability in log odds for placebo (Figure S8 (d)) but equal in log odds for active arms, respectively. In terms of scenarios for $\tau^{2}$, we selected the predictive log-normal distributions *L*$N\left( -3.95, {1.34}^{2} \right)$ (median: 0.02; interquartile range: 0.01 – 0.04) and *L*$N\left( -2.56, {1.74}^{2} \right)$ (median: 0.08; interquartile range: 0.03 – 0.26) to reflect small and substantial $\tau^{2}$, respectively. These predictive distributions referred to the expected $\tau^{2}$ in a future meta-analysis for all-cause mortality and a generic healthcare setting, respectively. ^3^ Then, the event risks in each arm of every trial were back-calculated as follows:

$$p_{i,k}^{E}=\frac{1}{1+exp\left( -{logit}_{i,k}^{E} \right)}, k=N,O$$

$$p_{i,k}^{C}=\frac{1}{1+exp\left( {-logit}_{i,k}^{C} \right)}, k=O,P$$

for the experimental and control arm, respectively.

References

1. Hartung J, Knapp G. A refined method for the meta-analysis of controlled clinical trials with binary outcome. Stat Med. 2001;20(24):3875-3889.
2. Spineli LM. An empirical comparison of Bayesian modelling strategies for missing binary outcome data in network meta-analysis. BMC Med Res Methodol. 2019. In press. DOI: 10.1186/s12874-019-0731-y.
3. Turner RM, Jackson D, Wei Y, Thompson SG, Higgins JPT. Predictive distributions for between-study heterogeneity and simple methods for their application in Bayesian meta-analysis. Stat Med. 2015;34(6):984-998.
4. **Description of scenarios on imbalance of missing outcome data**

To capture the imbalance in MOD between the compared arms, we assumed placebo to have more MOD than the active arms following our previous empirical study (Figure S8(e)) and, in addition, we assumed old intervention to have more MOD in the old-controlled trials. Then we generated $q_{i,k}^{E}$ with $k=N,O$ as follows:

$$q_{i,k}^{E}\sim\left\{ \begin{aligned} \\ \begin{matrix} U\left( 0.05, 0.10 \right), & \text{moderate missingness} \end{matrix} \\ \begin{matrix} U\left( 0.21, 0.30 \right), & \text{large missingness} \end{matrix} \end{aligned} \right.$$

whereas we generated $q_{i,k}^{C}$ with $k=O,P$ from

$$q_{i,k}^{C}\sim\left\{ \begin{aligned} \\ \begin{matrix} U\left( 0.11, 0.20 \right), & \text{moderate missingness} \end{matrix} \\ \begin{matrix} U\left( 0.31, 0.40 \right), & \text{large missingness} \end{matrix} \end{aligned} \right.$$

Under moderate and large MOD, imbalances had a range of 1 – 15% (median: 8%, interquartile range: 6 – 10%) and 1 – 18% (median: 10%, interquartile range: 7 – 12%), respectively (also observed in our empirical study ^1^; Figure S8(f)).

References

1. Spineli LM. An empirical comparison of Bayesian modelling strategies for missing binary outcome data in network meta-analysis. BMC Med Res Methodol. 2019. In press. DOI: 10.1186/s12874-019-0731-y.
2. **Generating log IMORs**

We used the pattern-mixture model to indicate the outcome among the missing participants in each arm of every trial. Specifically, we assumed patients randomized in the new or old intervention to be twice more likely to dropout due to the improvement of their outcome as opposed to patients receiving placebo. Subsequently, for each trial, we generated initially positive and negative log IMORs for the active interventions and placebo, respectively, through the following truncated-normal distributions:

| $\varphi_{i,k}^{PM}\sim TN\left( \mu=log\left( 2 \right), \sigma^{2}=1,\alpha=log(1) \right), k=N,O$  $\varphi_{i,P}^{PM}\sim TN\left( \mu=-log\left( 2 \right), \sigma^{2}=1,\alpha=log(1) \right)$ | (1) |
| --- | --- |

to indicate a greater likelihood of missing an event and non-event, in the active interventions (new and old) and placebo, respectively. Note that $log(1)$ refers to the truncated values of the respective distributions. As the reference scenario, we assumed MAR on average and we generated log IMORs through the following normal distribution:

$$\varphi_{i,k}^{PM}\sim N\left( 0, 1 \right), k=N,O,P$$

1. **Formula for the probability of observed events**

Then, we used the linkage function as described in Turner et al. ^1^ (equation 7, there) to obtain the probability of events among the completers in each arm of every trial:

$$p_{i,k}^{E,obs}=\frac{-\left( \Gamma_{i,k}^{E}-1 \right)-\sqrt{\left( \Gamma_{i,k}^{E}-1 \right)^{2}-4\cdot p_{i,k}^{E}\cdot\left( 1-q_{i,k}^{E} \right)\cdot\left( 1-\delta_{i,k}^{PM} \right)}}{2\cdot\left( 1-q_{i,k}^{E} \right)\cdot\left( 1-\delta_{i,k}^{PM} \right)},k=N,O$$

$$p_{i,k}^{C,obs}=\frac{-\left( \Gamma_{i,k}^{C}-1 \right)-\sqrt{\left( \Gamma_{i,k}^{C}-1 \right)^{2}-4\cdot p_{i,k}^{C}\cdot\left( 1-q_{i,k}^{C} \right)\cdot\left( 1-\delta_{i,k}^{PM} \right)}}{2\cdot\left( 1-q_{i,k}^{C} \right)\cdot\left( 1-\delta_{i,k}^{PM} \right)},k=O,P$$

with $\Gamma_{i,k}^{E}=\left( q_{i,k}^{E}-p_{i,k}^{E} \right)\cdot\left( 1-\delta_{i,k}^{PM} \right)$, $k=N,O$ and $\Gamma_{i,k}^{C}=\left( q_{i,k}^{C}-p_{i,k}^{C} \right)\cdot\left( 1-\delta_{i,k}^{PM} \right)$, $k=O,P$ for the experimental and control arm, respectively.

Reference

1. Turner NL, Dias S, Ades AE, et al. A Bayesian framework to account for uncertainty due to missing binary outcome data in pairwise meta-analysis. Stat Med. 2015;34(12):2062-2080.
2. **Probability of being best under pattern-mixture model: informative MOD**


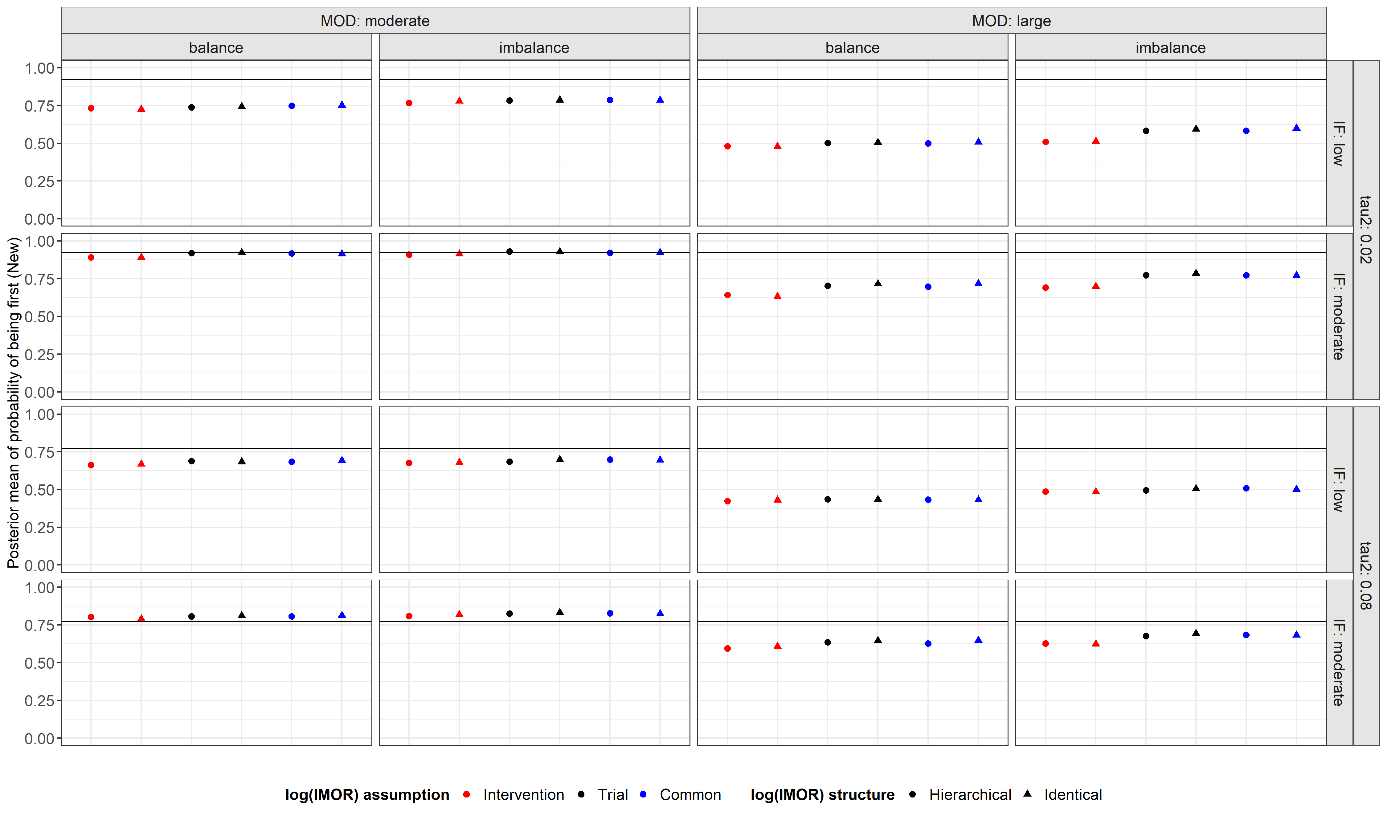


**Figure S9.** Posterior distribution of probability of being best for the new intervention under informative missing outcome data while using pattern-mixture model and accounting for the extent of missing outcome data (moderate, large), balance of missing outcome data (balance, imbalance), extent of inconsistency (low, moderate) and extent of $\tau^{2}$ (small, substantial). The horizontal solid lines reflect the true probability of being best for the new intervention under small and substantial true $\tau^{2}$. IF, inconsistency factor; MOD, missing outcome data.

1. **Simulation results under selection model: informative MOD**


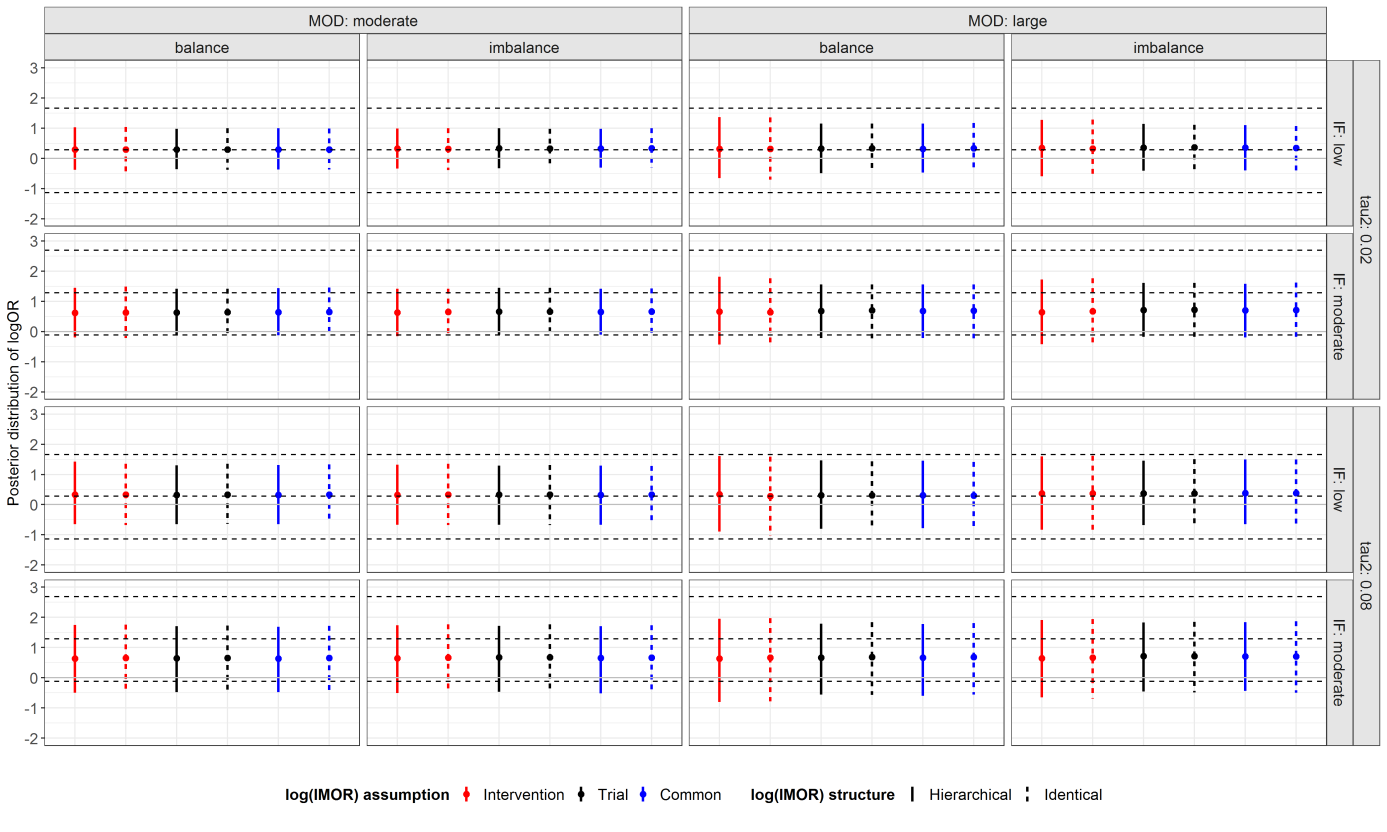


**Figure S10.** Posterior distribution of log OR (between new and old intervention) under informative missing outcome data while using selection model and accounting for the extent of missing outcome data (moderate, large), balance of missing outcome data (balance, imbalance), extent of $\tau^{2}$ (small, substantial) and extent of inconsistency (low, moderate). The horizontal dotted lines reflect the 95% interval and mean of the simulated distribution of log OR under low and moderate true inconsistency. IF, inconsistency factor; MOD, missing outcome data.


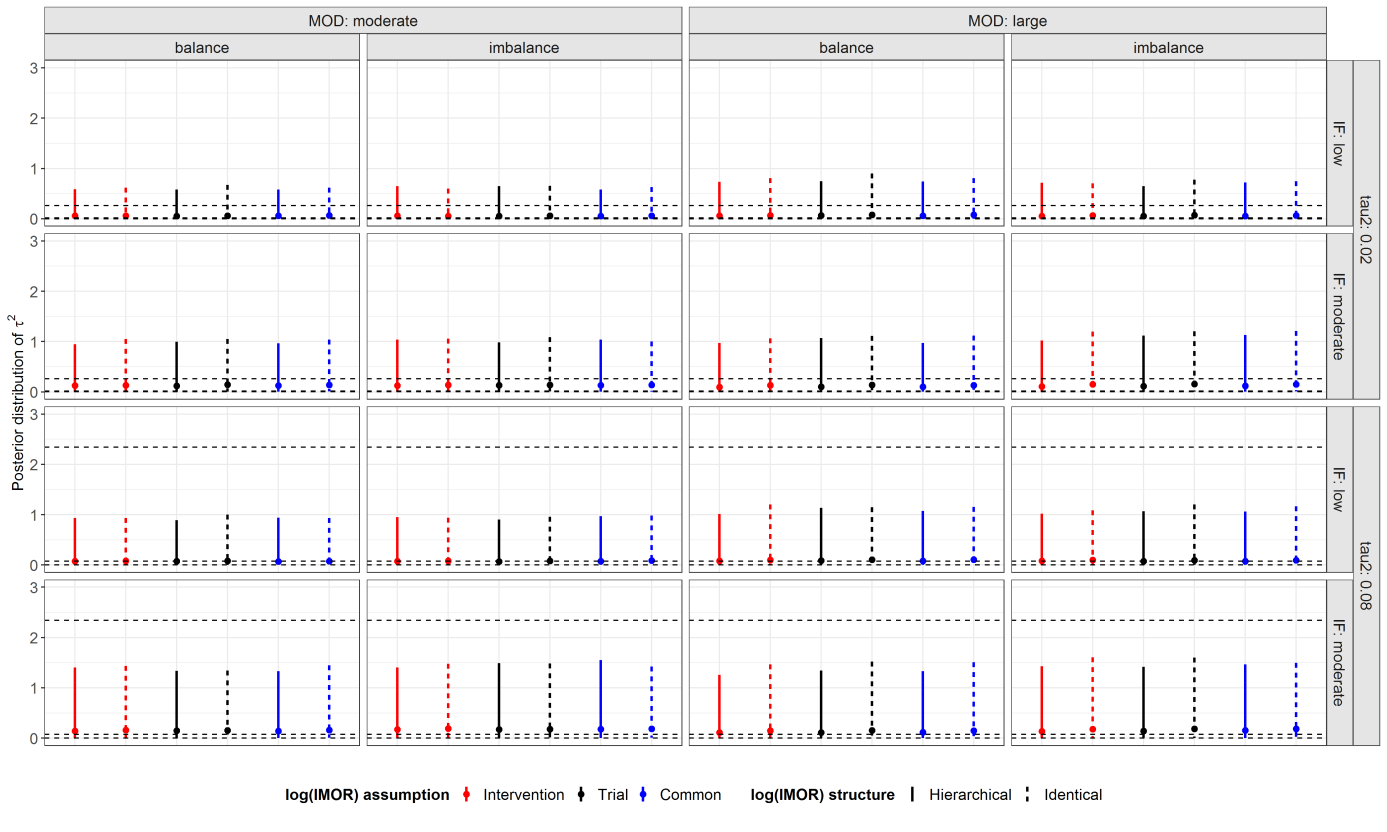


**Figure S11.** Posterior distribution of $\tau^{2}$ under informative missing outcome data while using selection model and accounting for the extent of missing outcome data (moderate, large), balance of missing outcome data (balance, imbalance), extent of $\tau^{2}$ (small, substantial) and extent of inconsistency (low, moderate). The horizontal dotted lines reflect the 95% interval and median of the simulated distribution of small and substantial $\tau^{2}$. IF, inconsistency factor; MOD, missing outcome data.


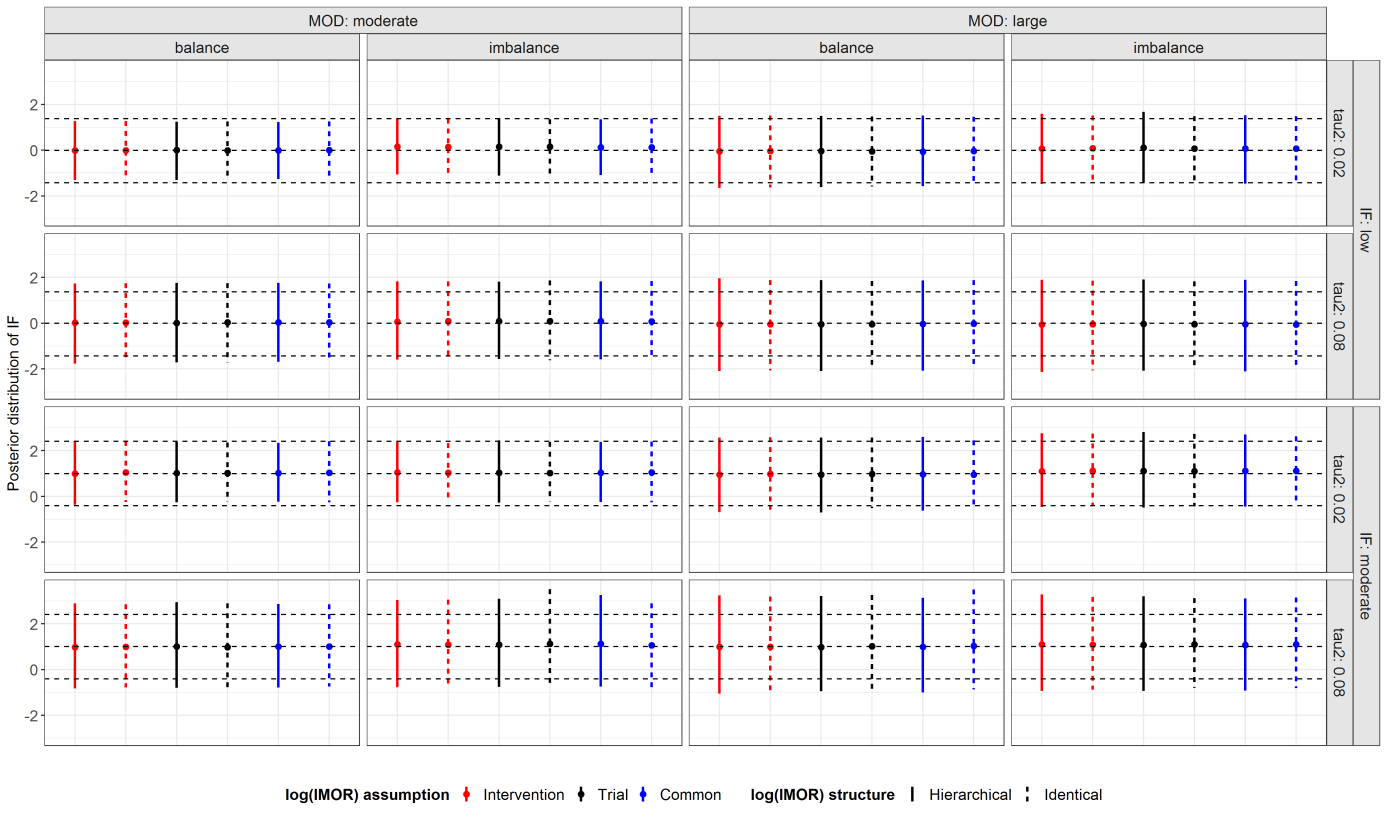


**Figure S12.** Posterior distribution of inconsistency factor (IF) under informative missing outcome data while using selection model and accounting for the extent of missing outcome data (moderate, large), balance of missing outcome data (balance, imbalance), extent of $\tau^{2}$ (small, substantial) and extent of inconsistency (low, moderate). The horizontal dotted lines reflect the 95% interval and mean of the simulated distribution of low and moderate IF. MOD, missing outcome data.


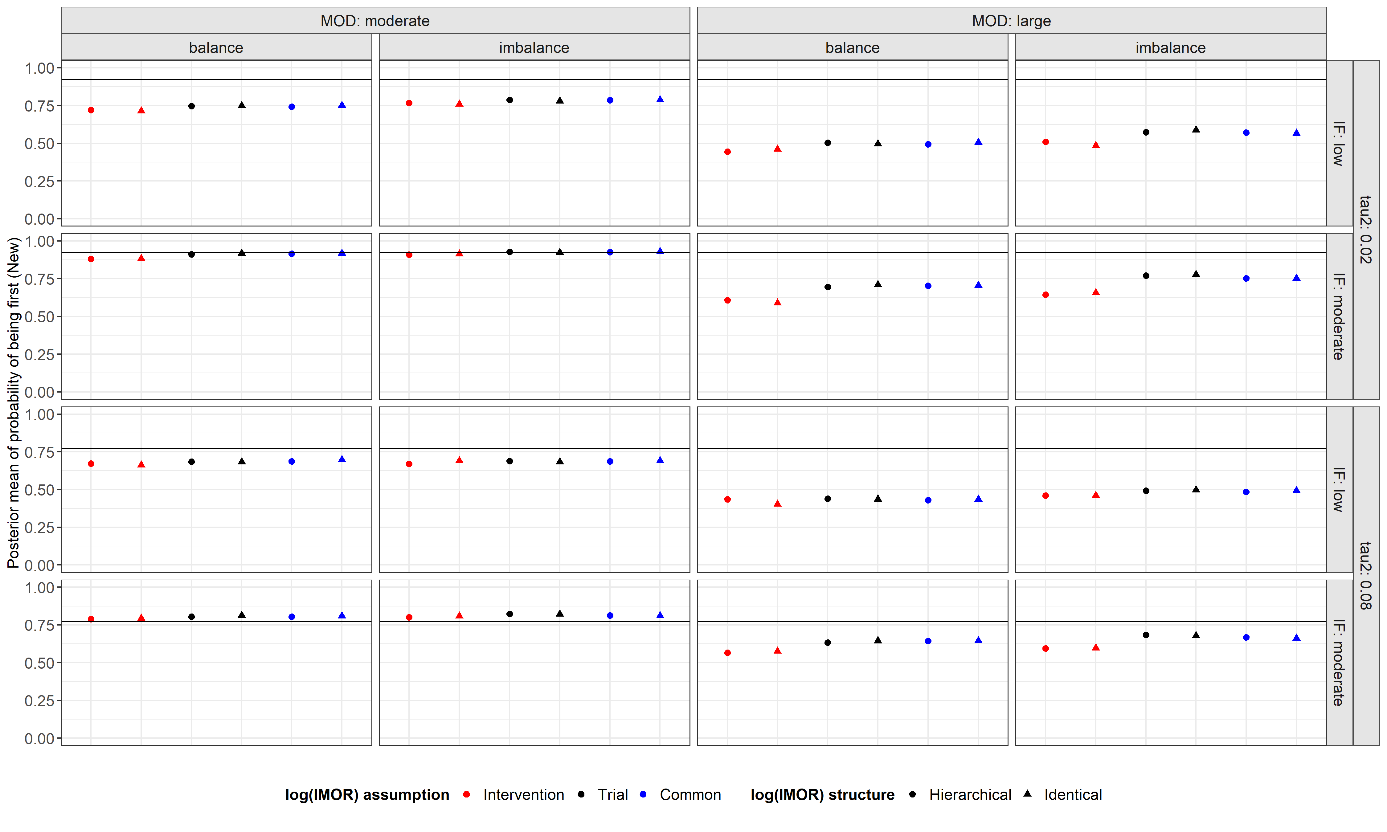


**Figure S13.** Posterior distribution of probability of being best for the new intervention under informative missing outcome data while using selection model and accounting for the extent of missing outcome data (moderate, large), balance of missing outcome data (balance, imbalance), extent of inconsistency (low, moderate) and extent of $\tau^{2}$ (small, substantial). The horizontal solid lines reflect the true probability of being best for the new intervention under small and substantial true $\tau^{2}$. IF, inconsistency factor; MOD, missing outcome data.

1. **Simulation results under pattern-mixture and selection models: MAR MOD**


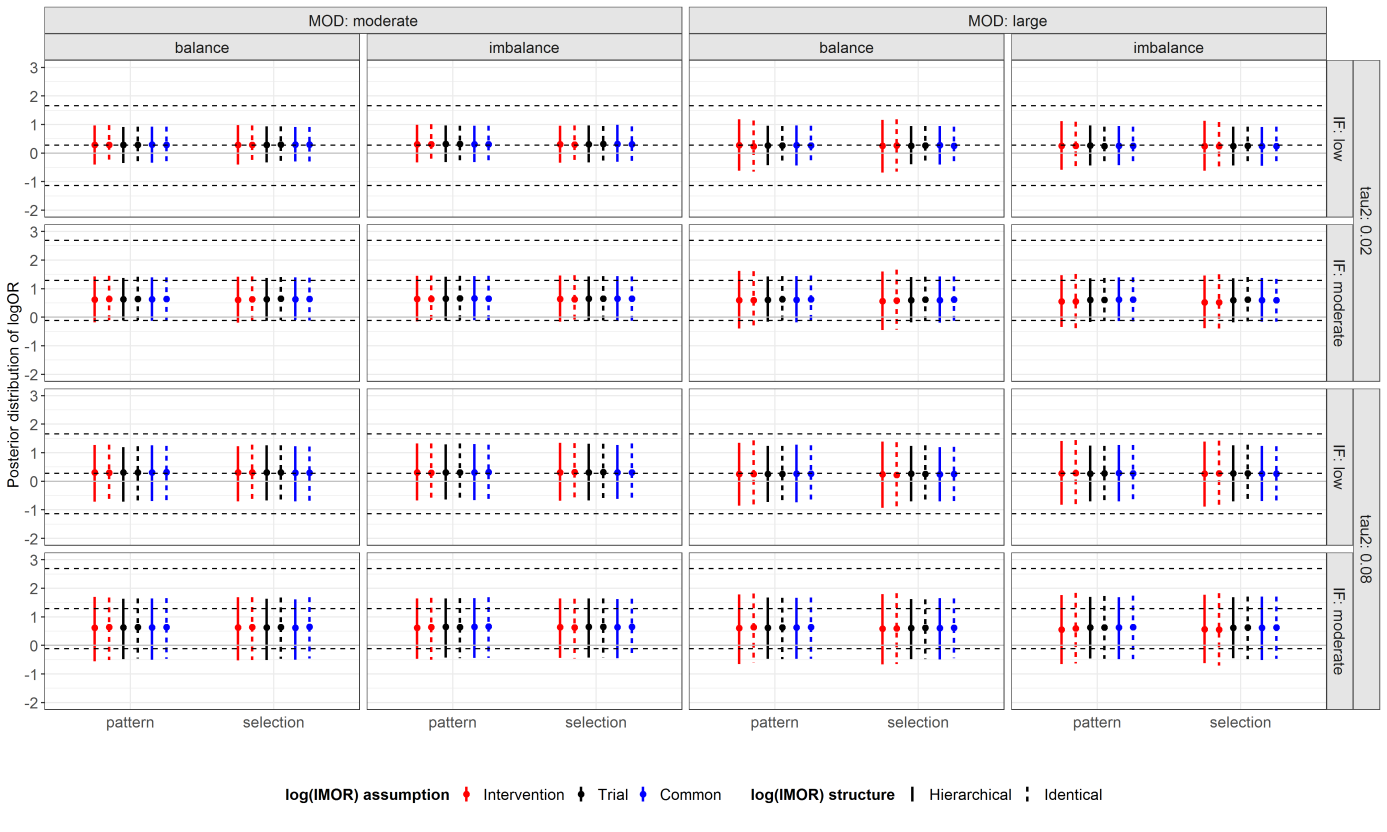


**Figure S14.** Posterior distribution of log OR (between new and old intervention) under missing at random while accounting for the model of missing outcome data (pattern-mixture and selection models), extent of missing outcome data (moderate, large), balance of missing outcome data (balance, imbalance), extent of $\tau^{2}$ (small, substantial) and extent of inconsistency (low, moderate). The horizontal dotted lines reflect the 95% interval and mean of the simulated distribution of log OR under low and moderate true inconsistency. IF, inconsistency factor; MOD, missing outcome data.


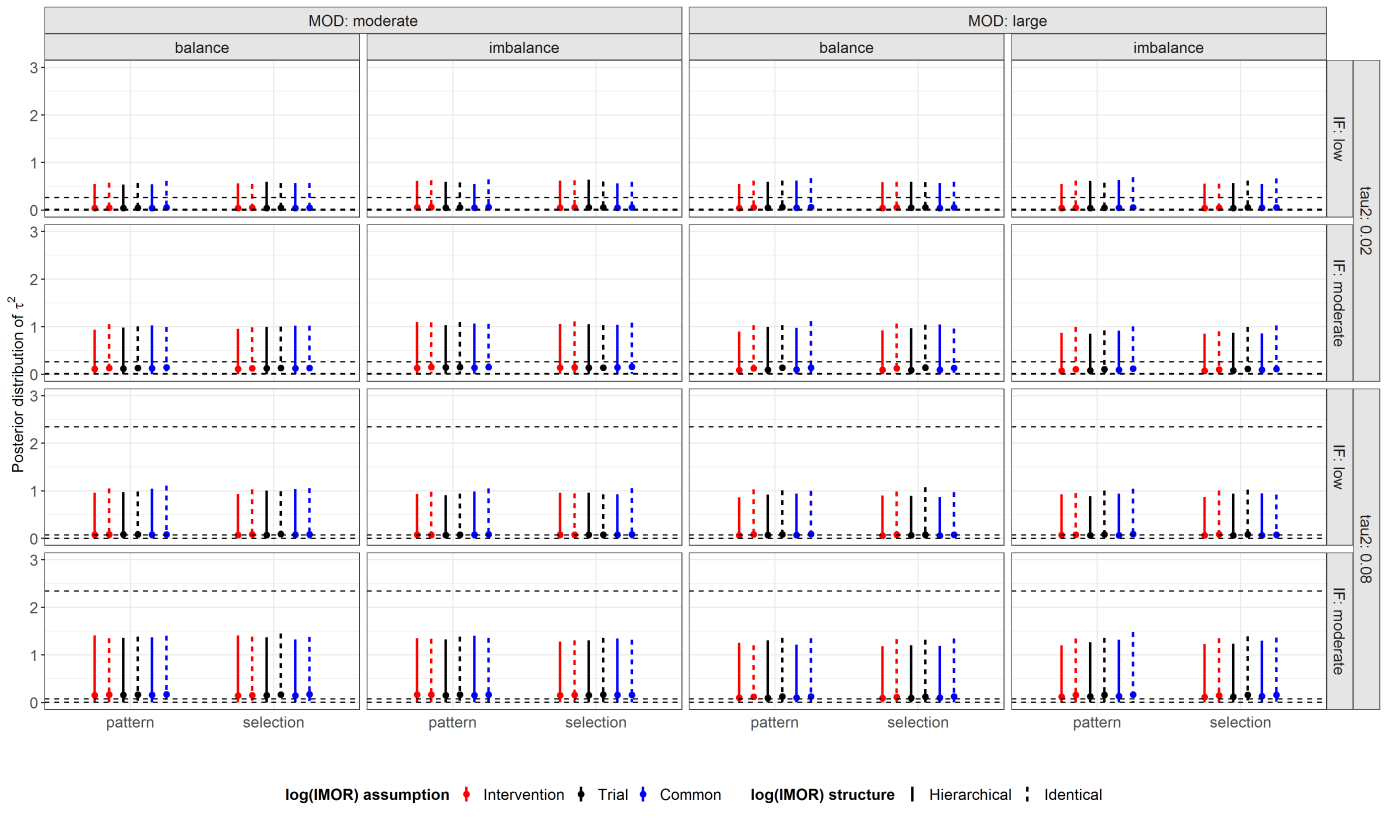


**Figure S15.** Posterior distribution of $\tau^{2}$ under missing at random while accounting for the model of missing outcome data (pattern-mixture and selection models), extent of missing outcome data (moderate, large), balance of missing outcome data (balance, imbalance), extent of $\tau^{2}$ (small, substantial) and extent of inconsistency (low, moderate). The horizontal dotted lines reflect the 95% interval and median of the simulated distribution of small and substantial $\tau^{2}$. IF, inconsistency factor; MOD, missing outcome data.


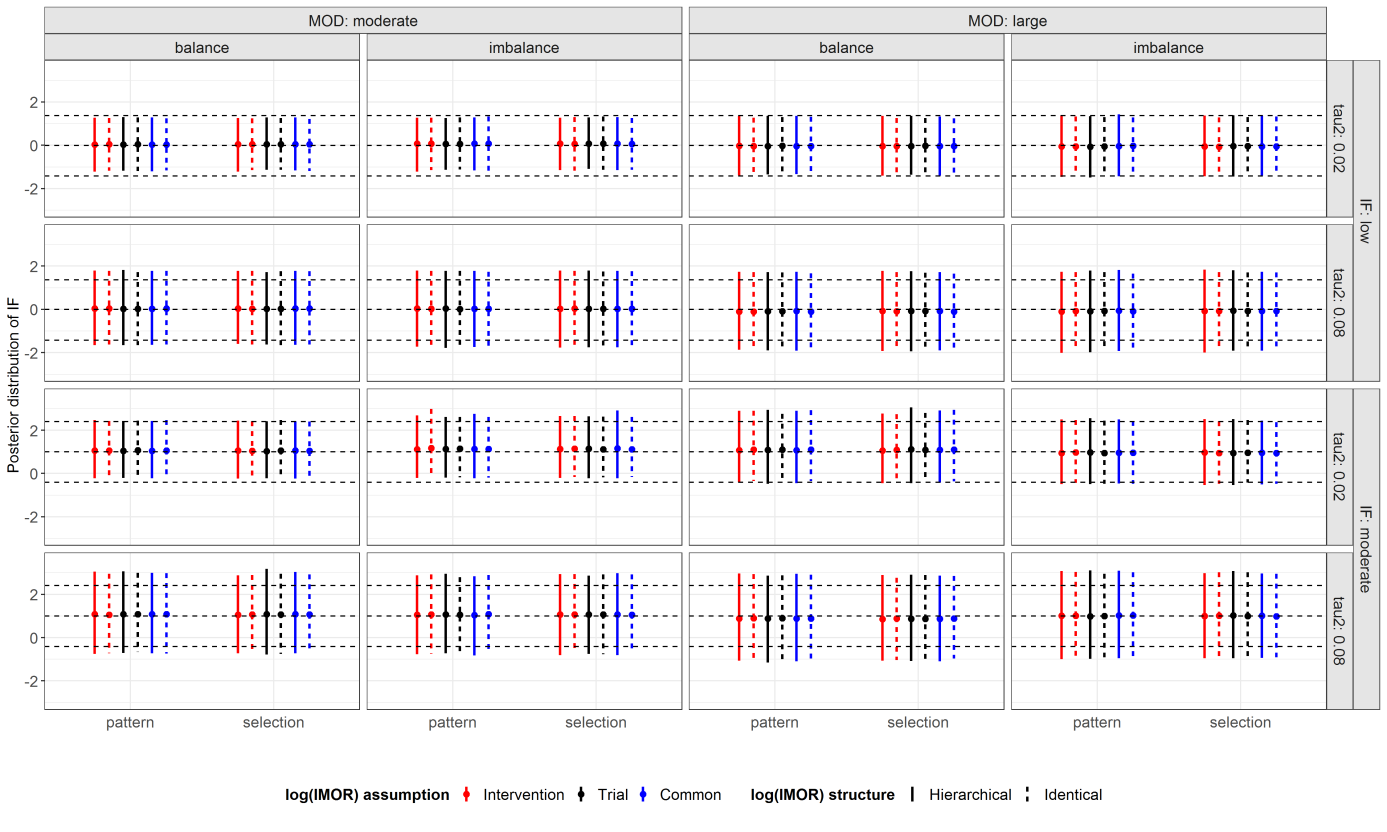


**Figure S16.** Posterior distribution of inconsistency factor (IF) under missing at random while accounting for the model of missing outcome data (pattern-mixture and selection models), extent of missing outcome data (moderate, large), balance of missing outcome data (balance, imbalance), extent of $\tau^{2}$ (small, substantial) and extent of inconsistency (low, moderate). The horizontal dotted lines reflect the 95% interval and mean of the simulated distribution of low and moderate IF. MOD, missing outcome data.


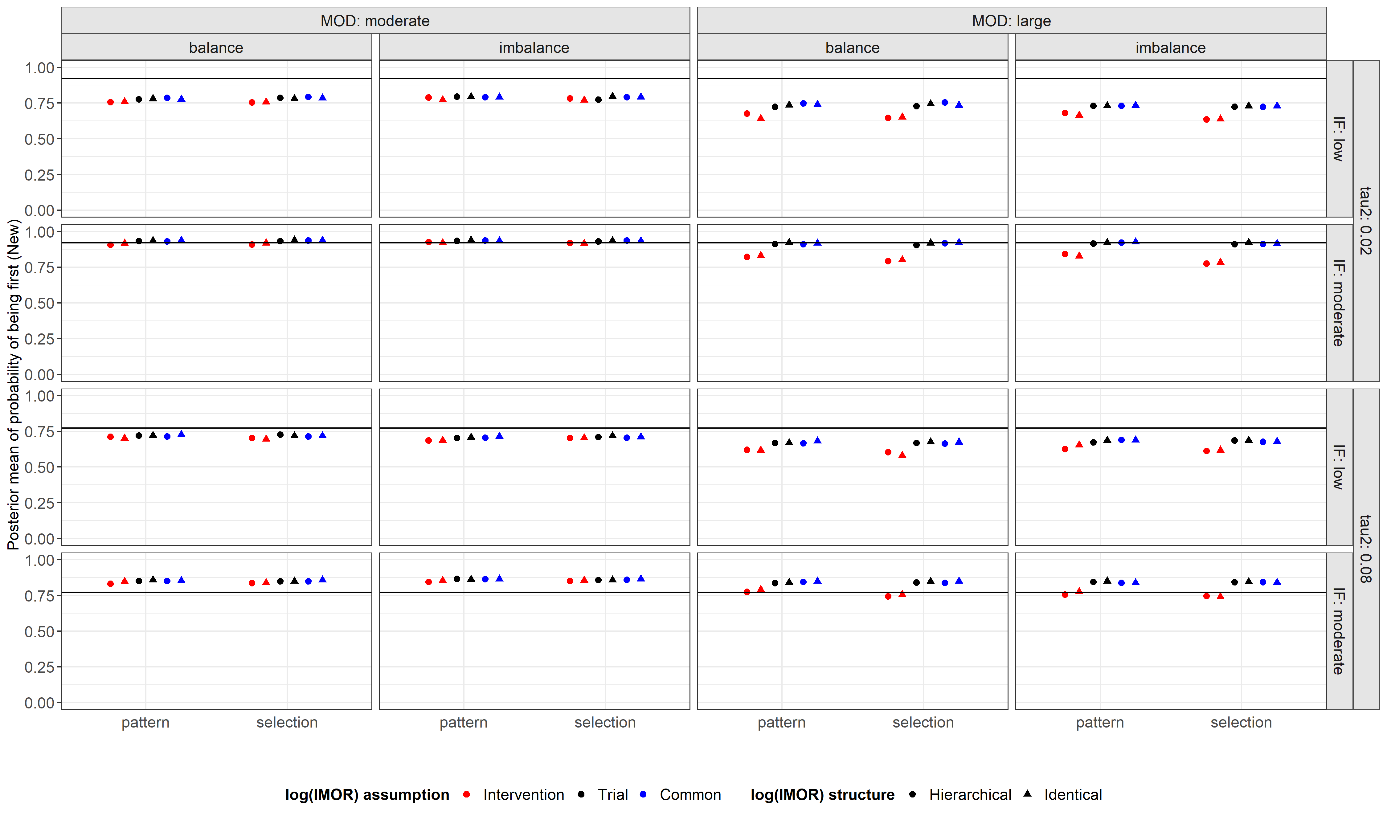


**Figure S17.** Posterior distribution of probability of being best for the new intervention under missing at random while accounting for the model of missing outcome data (pattern-mixture and selection models), extent of missing outcome data (moderate, large), balance of missing outcome data (balance, imbalance), extent of inconsistency (low, moderate) and extent of $\tau^{2}$ (small, substantial). The horizontal solid lines reflect the true probability of being best for the new intervention under small and substantial true $\tau^{2}$. IF, inconsistency factor; MOD, missing outcome data.
